# Bracing for the storm: emerging resistance mechanisms to KRAS inhibitors in pancreatic cancer and strategies to overcome them

**DOI:** 10.20517/cdr.2026.52

**Published:** 2026-08-31

**Authors:** Marco Pagano Mariano, Enrica Sgarilli, Dirk Mijnlieff, Bonifiya Visuvasam, Andrea Blesio, Graziana Digiacomo, Andrea Cavazzoni, Manuela Ferracin, Elisa Giovannetti

**Affiliations:** ^1^Department of Medical and Surgical Sciences, University of Bologna, Bologna 40126, Italy.; ^2^Department of Medicine and Surgery, University of Parma, Parma 43126, Italy.; ^3^Department of Medical Oncology, Cancer Center Amsterdam, Amsterdam UMC, Vrije Universiteit, Amsterdam 1081 BT, the Netherlands.; ^4^Medical Oncology, IRCCS Azienda Ospedaliero-Universitaria di Bologna, Bologna 40126, Italy.; ^5^Cancer Pharmacology Lab, Fondazione Pisana per la Scienza, San Giuliano 56017, Italy.; ^#^Authors contributed equally.

**Keywords:** PDAC, G12D, G12V, EMT, tumor microenvironment, autophagy, Hippo pathway, MAPK pathway

## Abstract

Oncogenic KRAS mutations are a defining feature of pancreatic ductal adenocarcinoma (PDAC), one of the most lethal solid malignancies, characterized by poor responsiveness to chemotherapy. Recent clinical successes of direct KRAS inhibitors in other cancer types have renewed interest in KRAS-directed therapy for PDAC. However, early clinical experience has revealed often short-lived responses, highlighting the rapid emergence of resistance and the need to understand the mechanisms limiting durable benefit. This review summarizes current knowledge on molecular resistance to RAS-directed inhibitors in PDAC, organized into five categories: intrinsic resistance, KRAS-dependent mechanisms, KRAS-independent bypass signaling, downstream pathway reactivation, and tumor microenvironment (TME)-mediated resistance. Evidence from PDAC models is integrated with insights from other KRAS-driven malignancies, particularly non-small cell lung cancer, where direct KRAS inhibition has been studied extensively. Collectively, resistance appears to arise from layered adaptive processes rather than single alterations, including secondary *KRAS* mutations, receptor tyrosine kinase-driven bypass signaling, reactivation of mitogen-activated protein kinase (MAPK) and phosphoinositide 3-kinase (PI3K) pathways, stromal-mediated protection, and reduced drug exposure. Notably, until recently, most evidence in PDAC has been indirect, while direct investigation of resistance mechanisms in KRAS-targeted settings has expanded rapidly only in recent years. Building on these advances, ongoing clinical trials increasingly explore rational combination strategies targeting upstream regulators, downstream effectors, and the TME. However, critical challenges persist, including patient selection, treatment sequencing, toxicity, and the lack of pharmacodynamic frameworks. Overcoming resistance will require mechanistically guided combinations, improved disease-specific models, and biomarker-driven adaptive strategies to ultimately achieve durable clinical benefits in PDAC.

## INTRODUCTION

Pancreatic ductal adenocarcinoma (PDAC) remains one of the most fatal malignancies, with a 5-year survival rate below 13%, and is characterized by late diagnosis, early metastasis, and substantial therapeutic resistance^[1]^. Despite advances in imaging and supportive care, treatment options for PDAC remain limited. Only a minority of patients present with resectable disease, and even following surgical resection, recurrence rates still exceed 80%^[1]^. For patients with locally advanced or metastatic disease, systemic chemotherapy with FOLFIRINOX or gemcitabine-nab-paclitaxel regimens remains the therapeutic backbone, but objective response rates (ORR) are modest and rarely durable^[2,3]^. Moreover, unlike many other solid tumors, PDAC shows limited responsiveness to targeted therapies or immunotherapies due to its dense desmoplastic stroma, poor vascularization, and an immunosuppressive tumor microenvironment (TME)^[4,5]^.

Genomic studies have established activating KRAS mutations as central drivers of pancreatic tumorigenesis, with mutations detected in more than 90% of PDAC cases in most cohorts, although reported frequency varies according to cohort composition^[6,7]^. The oncogenic KRAS protein sustains proliferation, metabolic reprogramming, and stromal activation, making it a rational therapeutic target^[8]^. For decades, KRAS was considered undruggable due to its high affinity for guanosine triphosphate (GTP)/guanosine diphosphate (GDP) and lack of exploitable binding pockets. This perception changed with the development of KRAS G12C inhibitors, such as sotorasib and adagrasib, which demonstrated clinically beneficial activity in lung cancer and proved that direct RAS inhibition is feasible^[9,10]^.

Although KRAS G12C mutations account for < 2% of PDAC, G12C inhibitors have been evaluated in PDAC^[11]^. Reported outcomes with these drugs remain modest, with ORR of around 20% and a median progression-free survival (PFS) ranging from 1.5 to 5 months^[12]^. These modest outcomes highlight the possibility of resistance mechanisms even in mutation-matched cases. Nevertheless, the development of KRAS G12C inhibitors has expanded the therapeutic landscape targeting KRAS to encompass multiple approaches, including pan-RAS(ON) inhibitors, RAS(OFF) inhibitors, novel covalent binders for G12D and G12V, and indirect KRAS pathway inhibitors targeting upstream and downstream effectors^[13]^.

**Figure 1:**
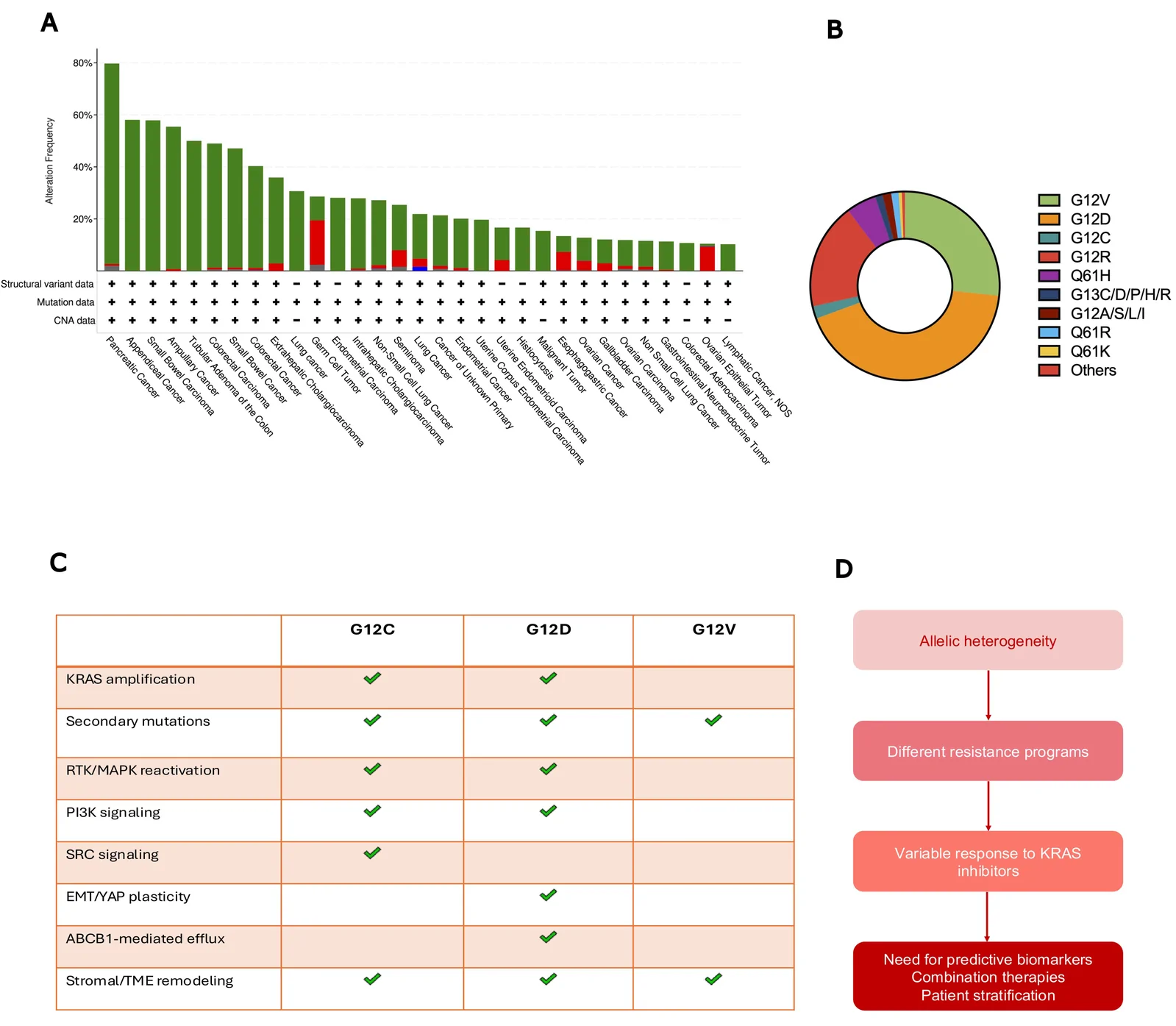
KRAS allelic heterogeneity and representative preclinical resistance mechanisms in PDAC. (A) Distribution of KRAS alterations across solid tumors with a mutation frequency of ≥ 10%. In this aggregated cBioPortal dataset, KRAS alterations were identified in 79.73% of 4,618 patients with PDAC; 77.09% corresponded to gene mutations, while the remaining cases included amplifications and multiple alterations. This value is lower than the > 90% KRAS mutation frequency commonly reported in dedicated PDAC cohorts, likely because aggregated datasets differ in cohort composition, tumor purity, sequencing depth, and definitions of KRAS mutation *vs.* broader genomic alteration. In colorectal carcinoma, KRAS was altered in 48.98% of 1,225 patients analyzed, with 47.76% representing gene mutations. In lung cancer, KRAS alterations were identified in 30.67% of cases, consistent with previously reported frequencies. The graph was generated using cBioPortal by combining 241 non-redundant studies; (B) Distribution of KRAS-mutant alleles in PDAC. G12D is the predominant KRAS alteration (~40%), followed by G12V (~35%) and G12R (~17%), whereas G12C is less frequent (~2%)^[11]^. Created in GraphPad Prism (v9.0); (C) Representative resistance mechanisms associated with KRAS G12C-, G12D-, and G12V-mutant PDAC reported in preclinical models. The mechanisms include KRAS copy-number gain, secondary mutations, RTK/MAPK reactivation, PI3K signaling, SRC signaling, EMT/YAP-mediated cellular plasticity, ABCB1-mediated drug efflux, and stromal/TME remodeling. Check marks indicate mechanisms reported in preclinical models carrying the indicated KRAS allele; blank cells indicate that no allele-specific evidence was identified in the reviewed literature ^[16,18,44,45,60,62,64,73,74,95,99]^. These mechanisms are not necessarily allele-exclusive, and clinical validation remains limited; (D) Conceptual representation of how KRAS allelic heterogeneity may contribute to distinct, partially overlapping resistance programs and potentially divergent responses to KRAS-targeted therapies, highlighting the need for predictive biomarkers, rational combination strategies, and patient stratification. PDAC: Pancreatic ductal adenocarcinoma; RTK: receptor tyrosine kinase; MAPK: mitogen-activated protein kinase; PI3K: phosphoinositide 3-kinase; SRC: proto-oncogene tyrosine-protein kinase Src; EMT: epithelial-to-mesenchymal transition; YAP: Yes-associated protein; ABCB1: ATP-binding cassette subfamily B member 1; TME: tumor microenvironment.

Across multiple KRAS-driven malignancies [Figure 1], resistance to KRAS inhibitors rarely follows a single path. Studies in non-small cell lung cancer (NSCLC) have shown that epithelial-to-mesenchymal transition (EMT), signal bypass, receptor tyrosine kinase (RTK) amplification, or secondary KRAS mutations may drive resistance, highlighting the complexity of adaptive responses to KRAS inhibition across tumor types^[14,15]^. In PDAC, one recent study identified stromal remodeling as a protective mechanism against KRAS inhibition^[16]^, while another study showed that the tumor is capable of adapting to the silencing of RAS downstream signals, such as extracellular signal-regulated kinase (ERK), via a feedback activation through phosphoinositide 3-kinase (PI3K)^[17]^. As KRAS acts as a signaling protein rather than a single linear effector, resistance often arises through several parallel mechanisms rather than a single dominant route^[18]^. For instance, a recent *in vivo* study in KPC mice (a genetically engineered model with pancreatic expression of oncogenic KRAS and mutant TP53) confirmed the KRAS dependency of pancreatic intraepithelial neoplasia^[19]^. Specifically, a pan-RAS inhibitor, RMC-7977, or a mutant-specific RAS inhibitor, RMC-9945, reduces preneoplastic lesion burden and delays tumor onset, increases cell death, silences mitogen-activated protein kinase (MAPK) signaling, and selectively alters the pre-TME^[19]^. However, tumor suppression varied by compound; RMC-9945 is more effective, but early-stage disease is less complex and plastic and is unable to rapidly compensate for the loss of signal caused by RAS inhibitors. The study highlights that these compounds may also be useful in early interception and that invasive PDAC represents a more complex challenge, yet remains KRAS-dependent^[19]^.

Together, these limitations suggest that KRAS inhibitors will likely require combination strategies to achieve durable responses; therefore, understanding how PDAC escapes KRAS inhibition is essential for designing more effective therapies. However, maximizing pathway suppression must be balanced against the risk of increased systemic toxicity associated with multi-node inhibition. Consequently, future therapeutic approaches will likely require biomarker-guided combinations, optimized sequencing strategies, and pharmacodynamically guided dosing schedules to improve both efficacy and feasibility.

This review aims to provide a PDAC-focused framework for understanding resistance to direct KRAS inhibition. We integrate evidence generated in PDAC models with insights from other KRAS-mutant tumor types, particularly colorectal cancer (CRC) and NSCLC, while distinguishing, whenever possible, PDAC-specific data from preclinical evidence and cross-cancer extrapolations. The goal is to identify the major biological and translational knowledge gaps that may limit the long-term success of KRAS-targeted therapy in PDAC and to discuss how these mechanisms can inform rational combination strategies, predictive biomarkers, and adaptive clinical trial designs.

## KRAS INHIBITORS AS THERAPY

Despite significant advances in systemic therapy, the standard first-line treatment for PDAC is based on cytotoxic chemotherapy regimens. In patients with good performance status, FOLFIRINOX or gemcitabine plus nab-paclitaxel provides the best survival benefit, as demonstrated in the PRODIGE and MPACT trials, although median overall survival (OS) rarely exceeds 11-12 months^[2,20]^. Unlike other solid tumors, PDAC has not shown responsiveness to targeted agents or immunotherapies, primarily due to its dense stroma, sparse vasculature, and profoundly immunosuppressive TME^[16,21]^. Given that over 90% of PDAC tumors harbor activating KRAS mutations, KRAS-directed therapy has long been considered a critical unmet need. Until recently, KRAS was viewed as undruggable, but advances have enabled the development of several new inhibitor classes that target either specific KRAS alleles or broader RAS signaling states [Table 1].

**Table 1:** Ongoing clinical trials for precise KRAS-directed therapies across solid tumors, including but not limited to PDAC

**ClinicalTrials.gov ID**	**Phase**	**Status**	**Enrollment**	**Treatment arm(s)**	**Combination regimen**	**Cohort**	**Primary outcome/end point**
KRAS G12C inhibitors							
NCT03600883	I/II	Recruiting	713 - Actual	Sotorasib (AMG 510)	Anti PD-1/L1; Midazolam	Advanced solid tumors including PDAC	AEs, DLTs, ORR
NCT03785249	I/II	Active, not recruiting	731 - Actual	Adagrasib (MRTX849)	Pembrolizumab; Cetuximab; Afatinib	Advanced solid tumors including PDAC	AEs, ORR
NCT05009329	I/II	Active, not recruiting	315 - Actual	Glecirasib (JAB-21822)	N/A	Advanced solid tumors including PDAC	AEs, DLTs, ORR
NCT04956640	I/II	Active, not recruiting	540 - Estimated	Olomarasib (LY3537982)	Pembrolizumab; Cetuximab; Pemetrexed; Cisplatin; Carboplatin	Advanced solid tumors including PDAC	AEs, DLTs antitumor activity
NCT04699188	I/II	Active, not recruiting	344 - Actual	Opnurasib (JDQ443)	TNO155 (SHP2 inhibitor), tislelizumab	Advanced solid tumors including PDAC	AEs, DLTs, ORR
NCT04585035	I/II	Active, not recruiting	180 - Actual	Garsorasib (D-1553)	Pembrolizumab, cetuximab	Advanced solid tumors including PDAC	AEs, DLTs
NCT06244771 (PROSPER)	I/II	Recruiting	403 - Estimated	FMC-376	N/A	Advanced solid tumors including PDAC	AEs, DLTs
NCT04449874	I	Active, not recruiting	498 - Estimated	Divarasib (GDC-6036)	Atezolizumab; Cetuximab; Bevacizumab; Erlotinib; GDC-1971 (SHP2 inhibitor); Inavolisib	Advanced solid tumors including PDAC	AEs, DLTs
NCT05462717	I	Active, not recruiting	222 - Estimated	Elironrasib (RMC-6291)	N/A	Advanced solid tumors including PDAC	AEs, DLTs
NCT04973163	I	Active, not recruiting	30 - Actual	BI 1823911	BI 1701963 (SOS1 inhibitor), Midazolam	Advanced solid tumors including PDAC	DLTs, ORR
NCT06128551	I/II	Recruiting	534 - Estimated	Elironrasib (RMC-6291)	Daraxonrasib (RMC-6236)	Advanced solid tumors including PDAC	AEs, ORR, DLTs
KRAS G12D inhibitors							
NCT05382559	I	Recruiting	681 - Estimated	ASP3082 (Setidegrasib)	Cetuximab, Leucovorin, Oxaliplatin, Fluorouracil, Irinotecan, Paclitaxel, Gemcitabine, Docetaxel, Pembrolizumab, Cisplatin, Carboplatin, Pemetrexed	Advanced solid tumors including PDAC	AEs, DLTs
NCT07483983	II	Recruiting	60 - Estimated	ASP3082 (Setidegrasib)	N/A	PDAC, non-small-cell lung cancer	DOR, CBR, PFS, OS, AEs, TEAEs, SAEs
NCT06040541	I	Recruiting	604 - Estimated	Zoldonrasib (RMC-9805)	Daraxonrasib (RMC-6236)	Advanced solid tumors including PDAC	AEs, DLTs
NCT07621718 (RASolute 305)	III	Recruiting	670 - Estimated	Zoldonrasib (RMC-9805)	Oxaliplatin, Leucovorin, 5-fluorouracil, Irinotecan, Gemcitabine, Nab-paclitaxel	Metastatic PDAC	PFS, OS
NCT05533463	I	Active, not recruiting	102 - Actual	HRS-4642	N/A	Advanced solid tumors including PDAC	AEs, DLTs
NCT07020221	I/II	Recruiting	295 - Estimated	GFH375 (VS-7375)	Cetuximab, Carboplatin/Pemetrexed/Pembrolizumab, Gemcitabine/Nab-Paclitaxel, Gemcitabine	Advanced solid tumors including PDAC	AEs, TEAEs, TRAEs, SAEs, DLTs, ORR, PFS rate, PR and CR rates, DCR, DOR, and PFS
NCT07303465	II	Recruiting	60 - Estimated	RNK08954	N/A	PDAC	PFS
NCT06818812	I	Active, not recruiting	30 - Actual	INCB186748	Cetuximab, GEMNabP, mFOLFIRINOX	Advanced solid tumors including PDAC	DLTs, TEAEs
NCT06179160	I	Recruiting	710 - Estimated	INCB161734	Cetuximab, Retifanlimab, GEMNabP, mFOLFIRINOX, FOLFOX, INCA33890	Advanced solid tumors including PDAC	DLTs, TEAEs
NCT06586515	I	Recruiting	630 - Estimated	LY3962673	Cetuximab, Gemcitabine, nab-paclitaxel, Oxaliplatin, leucovorin, Irinotecan, 5-fluorouracil	PDAC, NSCLC, CRC	ORR, BOR, DOR, TTR, DCR, PK, Tmax, AUC
KRAS G12V inhibitors							
NCT07349537	I	Recruiting	574 - Estimated	RMC-5127	Daraxonrasib, cetuximab	Advanced solid tumors including PDAC	AEs, DLTs

PDAC: Pancreatic ductal adenocarcinoma; AEs: adverse events; DLTs: dose-limiting toxicities; ORR: objective response rate; N/A: not applicable; SHP2: Src homology 2 domain-containing protein tyrosine phosphatase 2; SOS1: son of sevenless 1; CBR: clinical bnefit rate; PFS: progression-free survival; OS: overall survival; TEAEs: treatment-emergent adverse events; SAEs: serious adverse events; TRAEs: treatment-related adverse events; PR: partial response; CR: complete response; DCR: disease control rate; DOR: duration of response; NSCLC: non-small cell lung cancer; CRC: colorectal cancer; BOR: best overall response; TTR: time to response; PK: pharmacokinetics; Tmax: time to maximum concentration; AUC: area under the curve.

**Figure 2:**
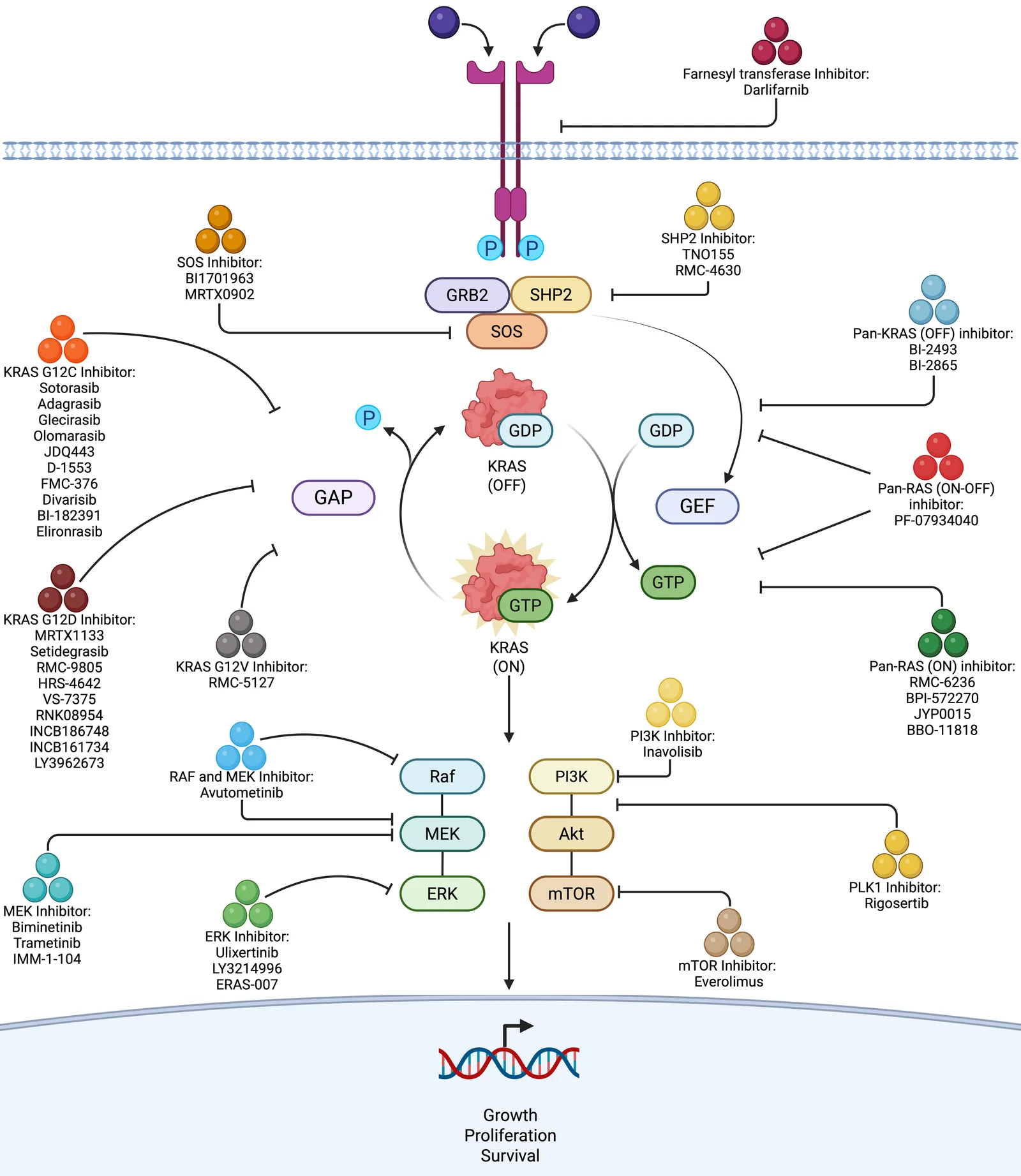
Schematic overview of KRAS signaling and its inhibitors in PDAC. KRAS, a GTPase, functions as a molecular switch between GDP- and GTP-bound states to drive RAF–MEK–ERK and PI3K–AKT–mTOR signaling, promoting cell proliferation and survival through transcriptional programs in the nucleus. The figure highlights inhibitors targeting the KRAS pathway at multiple levels, including upstream regulators, KRAS activation (ON and OFF states), its membrane localization, and downstream signaling effectors. Created in BioRender. Giovannetti, E. (2026) https://BioRender.com/u3f3uc9. PDAC: Pancreatic ductal adenocarcinoma; GDP: guanosine diphosphate; GTP: guanosine triphosphate; RAF: rapidly accelerated fibrosarcoma; MEK: mitogen-activated protein kinase kinase; ERK: extracellular signal-regulated kinase; PI3K: phosphoinositide 3-kinase; AKT: AKT serine/threonine kinase; mTOR: mechanistic target of rapamycin; GRB2: growth factor receptor-bound protein 2; SHP2: Src homology 2 domain-containing protein tyrosine phosphatase 2; SOS: son of sevenless; GAP: GTPase-activating protein; GEF: guanine nucleotide exchange factor; PLK1: Polo-like kinase 1.

KRAS is a small GTPase that belongs to the RAS family of signal-transducing proteins and functions as a central molecular switch downstream of RTKs, cycling between an inactive GDP-bound state and an active GTP-bound state to regulate key proliferative and survival pathways, including the RAF–MEK–ERK and PI3K–AKT–mTOR signaling pathways [Figure 2]. This cycling is tightly controlled by guanine nucleotide exchange factors (GEFs), which promote GTP loading, and GTPase-activating proteins (GAPs), which in turn accelerate GTP hydrolysis. In its active conformation, KRAS engages multiple effector proteins through its conserved switch I (residues 30-38) and switch II (residues 59-67) regions, rendering its activity highly sensitive to alterations in nucleotide binding and conformational dynamics^[22]^. Oncogenic KRAS mutations impair intrinsic and GAP-mediated GTP hydrolysis, thereby locking the protein in a signaling-competent state. In PDAC, mutations are highly enriched at codon 12 within the phosphate-binding loop (P-loop), most commonly G12D, G12V, and G12R, with less frequent alterations at codons 13 and 61^[11]^. These codons lie near the nucleotide-binding pocket and the switch II region, accounting for both their strong oncogenic potential and their relevance to drug binding. These substitutions not only drive constitutive pathway activation but also shape the structural availability for therapeutic targeting, thus influencing inhibitor binding, nucleotide cycling, and susceptibility to on-target resistance mechanisms^[11,23]^.

The first successful KRAS-targeted drugs were the KRAS G12C inhibitors sotorasib and adagrasib. These compounds bind covalently to the mutant cysteine in the switch-II pocket, locking KRAS in its inactive GDP-bound state. Although KRAS G12C occurs in only ~2% of PDAC, both inhibitors have been evaluated clinically^[11]^. Strickler *et al.* reported an ORR of 21% and a median PFS of 4 months in patients with PDAC treated with sotorasib^[12]^. More recently, Bekaii-Saab *et al.* obtained an ORR of 33% and a median PFS of 5.4 months with adagrasib in a similar patient population^[23]^. Responses are modest, but these data reflect that direct KRAS inhibition is feasible in PDAC.

KRAS G12D is the most prevalent mutation in PDAC (~40%), followed by KRAS G12V (~35%)^[11]^. Cysteine at position 12 represents an essential residue for the binding of sotorasib and adagrasib, whereas distinct compounds have been developed for other allelic variants^[24,25]^.

One of the most advanced therapies involves MRTX1133, a potent non-covalent KRAS G12D inhibitor with picomolar binding affinity. Multiple preclinical studies show specific and rapid antitumor activity in KRAS G12D models. In particular, MRTX1133 induced tumor-volume regression (*P* < 0.0001) in subcutaneous KRAS G12D immunocompetent PDAC models^[26]^. In addition, Mahadevan *et al*. demonstrated that MRTX1133 effectively inhibits the proliferation of human PDAC cells harboring KRAS G12D mutations^[27]^. As reported in Table 1, for the KRAS G12V mutation, RMC-5127 has advanced to phase I clinical evaluation. In contrast, for KRAS G12R, the third most common KRAS alteration in PDAC, no selective direct inhibitors have yet entered clinical practice. The arginine substitution at codon 12 induces distinct structural changes in KRAS, altering the conformation of the switch II region and making it more challenging to develop high-affinity mutant-selective inhibitors than for other variants, such as G12C and G12D. Consequently, significant efforts are currently focused on computational drug discovery approaches to identify novel compounds capable of selectively targeting the KRAS G12R isoform^[28,29]^.

Beyond allele-specific inhibitors, several new approaches are being developed to target multiple RAS isoforms simultaneously, offering broader therapeutic coverage and reduced escape through compensatory RAS family members. One major approach involves pan-RAS(ON) inhibitors [Table 2]. These compounds recruit Cyclophilin A to form a ternary complex that occludes the RAS–RAF interaction surface, inhibiting MAPK pathway signaling independent of the specific RAS allele. By binding to conformational features shared across GTP-loaded KRAS, NRAS, and HRAS, these compounds prevent interaction with downstream effectors such as RAF kinases, thereby suppressing MAPK pathway signaling irrespective of the underlying KRAS allele. This mode of action distinguishes pan-RAS(ON) inhibitors from covalent allele-specific agents and enables broader pathway coverage in RAS-driven tumors. Compounds such as RMC-6236, RMC-7977, and BPI-572270 exemplify this strategy^[13,30]^. The development of novel inhibitors specifically targeting the most frequent mutations in PDAC, such as KRAS G12D, alongside pan-RAS agents, has expanded the eligible PDAC cohort, far exceeding the limited cohorts of early KRAS G12C OFF-state inhibitors. Preliminary early-phase clinical data have reported encouraging activity signals for emerging RAS-targeted agents in PDAC, although response durability remains uncertain. KRAS G12D-directed agents, including zoldonrasib/RMC-9805 (NCT06040541)^[31,32]^ and GFH375 (NCT06500676)^[33,34]^, have shown preliminary antitumor activity in early-phase studies, while the pan-RAS(ON) inhibitor daraxonrasib/RMC-6236 has reported ORRs of approximately 25%-29% and median PFS of 7.6-8.5 months in pretreated RAS-mutant PDAC cohorts^[35]^. These results remain preliminary and require validation in larger, prospectively defined clinical studies^[13]^. In addition, combining selective mutant inhibitors with pan-RAS agents may represent a viable strategy to counteract compensatory signaling mediated by wild-type RAS and thereby delay the emergence of resistance^[13]^.

**Table 2:** Clinical trials of pan-RAS inhibitors in PDAC and other solid tumors

**ClinicalTrial.gov ID**	**Phase**	**Status**	**Enrollment**	**Treatment arm(s)**	**Mechanism of action**	**Combination regimen**	**Cohort**	**RAS mutation status**	**Primary outcome/end point**
pan-RAS inhibitors									
NCT07252232 (RASolute 304)	III	Recruiting	500 - Estimated	Daraxonrasib (RMC-6236)	pan-RAS(ON) inhibitor	N/A	Resected PDAC	N/A	AEs, DFS, OS
NCT06625320	III	Active, not recruiting	500 - Actual	Daraxonrasib (RMC-6236)	pan-RAS(ON) inhibitor	Gemcitabine, nab-paclitaxel, Irinotecan, Liposomal irinotecan, 5-fluorouracil, leucovorin, Oxaliplatin	Metastatic PDAC	KRAS, NRAS or HRAS codons 12, 13, or 61	PFS, OS
NCT05379985	I/II	Recruiting	754 - Estimated	Daraxonrasib (RMC-6236)	pan-RAS(ON) inhibitor	N/A	Advanced solid tumors including PDAC	KRAS, NRAS or HRAS codons 12, 13, or 61	AEs, DLTs
NCT07491445 (RASolute 303)	III	Recruiting	900 - Estimated	Daraxonrasib (RMC-6236)	pan-RAS(ON) inhibitor	Gemcitabine, Nab-paclitaxel	Metastatic pancreatic adenocarcinoma	N/A	PFS, OS
NCT06445062	I/II	Recruiting	1130 - Estimated	Daraxonrasib (RMC-6236)	pan-RAS(ON) inhibitor	mFOLFOX6, bevacizumab, mFOLFIRINOX, cetuximab, gemcitabine, nab-paclitaxel, Zoldonrasib (RMC-9805)	CRC, PDAC, gastrointestinal cancer	RAS G12D mutation (Subprotocol D, E, F)	AEs, DLTs
NCT07435038	I/II	Not yet recruiting	120 - Estimated	BPI-572270	pan-RAS(ON) inhibitor	N/A	Advanced solid tumors including PDAC	KRAS, NRAS or HRAS codons 12, 13, or 61	DLT, RP2D, ORR
NCT06895031	I/II	Recruiting	210 - Estimated	JYP0015	pan-RAS(ON) inhibitor	N/A	Advanced solid tumors including PDAC	KRAS, NRAS or HRAS codons 12, 13, or 61	DLT, AEs, ORR
NCT06917079	I	Recruiting	665 - Estimated	BBO-11818	pan-KRAS(ON/OFF) inhibitor	Pembrolizumab, Platinum chemotherapy, Pemetrexed, Cetuximab, FOLFOX, NALIRIFOX, Gemcitabine, Paclitaxel	CRC, PDAC, NSCLC	KRAS G12A, G12C, G12D, G12S, or G12V	TEAEs, SAEs, DLTs
NCT06447662	I	Active, not recruiting	64 - Actual	PF-07934040	pan-KRAS(ON/OFF) inhibitor	Gemcitabine + Nab-paclitaxel, Cetuximab, FOLFOX + Bevacizumab, Pembrolizumab, Platinum-based Chemotherapy	NSCLC, CRC, PDAC	KRAS, any variant	AEs, DLT, ORR

RAS: RAS family of small GTPases; PDAC: pancreatic ductal adenocarcinoma; N/A: not applicable; AEs: adverse events; DFS: disease-free survival; OS: overall survival; PFS: progression-free survival; CRC: colorectal cancer; DLT: dose-limiting toxicity; RP2D: determination of RP2D in the expansion phase; ORR: objective response rate; NSCLC: non-small cell lung cancer; TEAEs: treatment-emergent adverse events; SAEs: serious adverse events.

These emerging strategies, including compounds targeting KRAS G12D or multiple RAS isoforms, represent a promising therapeutic advance in PDAC; however, as with other targeted therapies, resistance remains a major challenge, leading to responses that are often limited or short-lived.

Importantly, the clinical translation of these strategies is challenged not only by biological resistance but also by trial-design and patient-related factors. Many early-phase studies include heterogeneous populations of advanced solid tumors, with PDAC patients often representing a relatively small subgroup. Moreover, results from completed clinical studies have highlighted that promising preclinical combinations do not necessarily translate into meaningful clinical benefit. This issue may be particularly relevant in PDAC, where advanced disease is frequently associated with cancer-related cachexia, malnutrition, comorbidities, and poor performance status, which can limit tolerance to intensive combination regimens^[36,37]^.

In addition to cancer-related performance status, accumulating evidence shows that PDAC develops resistance through multiple, often co-existing mechanisms, rather than a single dominant pathway. For conceptual clarity, these mechanisms are organized into five broad and non-mutually exclusive categories: (1) intrinsic resistance to KRAS inhibition; (2) KRAS-dependent mechanisms; (3) KRAS-independent mechanisms; (4) downstream pathway reactivation; and (5) tumor-microenvironment-mediated resistance. This classification combines temporal and mechanistic criteria and is intended as a conceptual classification rather than a rigid biological separation, as several mechanisms may converge on shared signaling outputs or coexist within the same tumor. Importantly, the strength and source of evidence vary substantially across these categories. Therefore, Table 3 summarizes the major resistance mechanisms discussed in this review, specifying the tumor model, type of evidence, inhibition or pathway setting, and potential therapeutic implications. This distinction is particularly relevant because some mechanisms are supported by PDAC-specific clinical or preclinical data, whereas others are inferred from studies in other tumor models.

**Table 3:** Summary of putative resistance mechanisms to KRAS inhibition in PDAC and their therapeutic implications

**Resistance category**	**Resistance mechanism**	**Tumor type**	**Type of evidence**	**Target/inhibition**	**Potential therapeutic implication**
Intrinsic resistance	Co-occurring alterations: e.g., TP53, CDKN2A, and SMAD4 loss	Lung cancer	Indirect preclinical *in vivo* evidence for therapy response; PDAC genomic evidence for frequency	KRAS pathway inhibition/MEK inhibition	Co-mutation profiling may refine patient selection and improve treatment outcomes beyond KRAS status alone^[40,41]^
	Pre-existing RTK or downstream effector activation	Lung cancer, PDAC	Indirect preclinical lung cancer evidence; PDAC biological evidence	RAS inhibition; RAF, MEK, PI3K and AKT inhibition	Co-targeting RTKs or downstream effectors may enhance the efficacy of RAS inhibition^[38,42]^
	YAP/TAZ activation and Hippo pathway dysregulation, EMT-associated resistant state	Colon cancer, PDAC	Indirect preclinical colon cancer evidence; PDAC literature and clinical association evidence	KRAS suppression/silencing	Supports that baseline lineage states influence KRAS-targeted therapy sensitivity and that YAP/TAZ-associated transcriptional programs may represent therapeutic vulnerabilities^[43-46]^
	Preexisting secondary KRAS mutations	Multiple cancer types	Indirect literature evidence	Targeted therapy	Supports molecular profiling to guide targeted therapy selection and treatment adaptation^[47,48]^
KRAS-dependent resistance	KRAS amplification	Colon cancer, PDAC, NSCLC	Indirect and direct preclinical and clinical evidence across tumor types	KRAS inhibition	Supports KRAS amplification as an escape mechanism and rationale for broader RAS inhibition^[18,51,52]^
	Emergence of secondary KRAS mutations	Murine cells, lung cancer, CRC	Indirect preclinical evidence in murine and lung cancer cells; indirect clinical evidence on lung cancer and CRC	KRAS inhibition	Suggests that resistance may arise through reactivation of RAS pathway signaling despite continued drug exposure^[15,49,50]^
	Altered nucleotide cycling through SOS1/SHP2 activation	PDAC, lung cancer	Preclinical evidence	KRAS, MEK and SOS1/SHP2 inhibition	Supports combinations of KRAS inhibitors with SOS1 or SHP2 blockade^[53,54]^
	Impaired GAP activity and increased RAS-GTP loading	PDAC, NSCLC	Preclinical evidence	RAS nucleotide cycling/RAS-GTP effector engagement	Provides rationale to target RAS network dynamics rather than RAS mutation status alone^[55]^
KRAS-independent resistance	Adaptive reversible state	PDAC	Preclinical evidence	KRAS silencing, focal adhesion signaling	Supports targeting adhesion/focal adhesion signaling to enhance KRAS inhibition^[64]^
	Upstream RTK-driven bypass	PDAC, NSCLC, CRC	Indirect and direct preclinical evidence in KRAS-mutant models; extrapolated mechanistic evidence from BRAF V600E-driven models	KRAS, RTK, SHP2, MEK/ERK and MAPK pathway inhibition	Supports the rationale for vertical combination strategies^[60,61,62,64]^
	Wild-type RAS paralogue engagement (NRAS and HRAS)	PDAC, NSCLC	Literature evidence; indirect preclinical evidence	KRAS pathway; MEK inhibition	Supports pan-RAS inhibitors to limit compensation by wild-type RAS isoforms^[63,66]^
	ERBB3-mediated PI3K-AKT activation	Lung cancer, breast cancer	Indirect preclinical evidence	MEK inhibition	Supports the concept of adaptive rewiring after single-pathway inhibition through PI3K pathway reactivation^[65]^
	MET amplification bypass	NSCLC	Indirect preclinical evidence	KRAS inhibition, MET inhibition	Supports MET amplification as a potential bypass resistance mechanism to KRAS inhibition^[67]^
	Incomplete suppression of the PI3K-AKT pathway and adaptive reactivation of MAPK signaling	NSCLC	Indirect preclinical evidence	KRAS inhibition, PI3K inhibition	Supports the rationale for PI3K pathway co-targeting in RAS-mutant tumors^[39]^
	EGFR and c-MET expression associated with aggressive PDAC biology	PDAC	Clinical-prognostic evidence	N/A	Suggests the prognostic relevance of EGFR or c-MET upstream signaling in PDAC, although not directly linked to KRAS mutation status or KRAS inhibitor resistance^[68-70]^
	ABCB1-mediated drug efflux	PDAC/multiple cancer types	Indirect preclinical evidence	KRAS inhibition	Supports drug efflux as a resistance mechanism^[18,71,72]^
	SRC-mediated pathway rewiring	Multiple cancer types	Literature evidence	KRAS inhibition	Supports SRC activation as a potential resistance mechanism and rationale for co-targeting SRC^[73]^
Downstream pathway reactivation	MAPK pathway rebound	PDAC	Preclinical evidence	KRAS, MEK inhibition	Provides preclinical rationale for mTORC1/2 co-targeting to overcome adaptive resistance to KRAS inhibition in PDAC^[74]^
	Disease progression despite ERK inhibition	PDAC	Clinical evidence	ERK inhibition	Supports the concept that single-pathway inhibition (MAPK) may be insufficient to achieve durable disease control^[76-78]^
	Autophagy induction after MAPK suppression	PDAC	Indirect preclinical evidence and clinical trial	MEK/ERK inhibition	Supports the combination of MAPK pathway inhibitors with autophagy inhibition^[11,81-84]^
	MYC-RPIA-dependent flux maintenance despite KRAS inhibition	PDAC	Preclinical evidence	KRAS or MEK inhibition	Suggests metabolic co-targeting as a strategy to eliminate downstream functional escape^[85]^
	KRAS downstream metabolic rewiring involving glucose, glutamine/asparagine, and lipid metabolism despite KRAS inhibition	PDAC	Preclinical evidence	DON, L-asparaginase, MEK inhibition, fatty acid oxidation inhibition	Supports co-targeting metabolic dependencies and KRAS downstream signaling to maintain durable tumor suppression and overcome resistance^[80,83,84]^
TME mediated resistance	Stromal physical barrier limiting drug penetration	PDAC	Indirect clinical and preclinical evidence	N/A	Supports strategies improving drug delivery and stromal reprogramming^[86-94]^
	CAF-derived paracrine signaling	PDAC	Preclinical evidence	RAS/MAPK pathway inhibition	Provides the rationale for FAK inhibition or CAF reprogramming to reduce stromal-mediated adaptive resistance^[96-99]^
	Immune remodeling after KRAS inhibition	PDAC	Preclinical evidence	(K)RAS inhibition	Supports combinations with immune checkpoint blockade during a potential immunostimulatory window^[95,100]^
	YAP1-associated immunosuppressive TME remodeling	PDAC	Preclinical evidence	Pan-KRAS inhibition	Suggests combined targeting of tumor-cell plasticity and immune/stromal escape mechanisms^[95]^
	Stromal and immune-mediated resistance	PDAC	Preclinical and clinical evidence	FAK inhibition, ICIs, RAF/MEK inhibition	Supports stromal reprogramming strategies^[21,99,101,102]^

PDAC: Pancreatic ductal adenocarcinoma; MEK: mitogen-activated protein kinase kinase; RTK: receptor tyrosine kinase; RAS: RAS family of small GTPases; RAF: rapidly accelerated fibrosarcoma; PI3K: phosphoinositide 3-kinase; AKT: AKT serine/threonine kinase; YAP: Yes-associated protein; TAZ: transcriptional co-activator with PDZ-binding motif; EMT: epithelial-to-mesenchymal transition; NSCLC: non-small cell lung cancer; CRC: colorectal cancer; SOS1: son of sevenless 1; SHP2: Src homology 2 domain-containing protein tyrosine phosphatase 2; GAP: GTPase-activating protein; GTP: guanosine triphosphate; ERK: extracellular signal-regulated kinase; MAPK: mitogen-activated protein kinase; EGFR: epidermal growth factor receptor; MET: mesenchymal-epithelial transition factor; N/A: not applicable; ABCB1: ATP-binding cassette subfamily B member 1; SRC: proto-oncogene tyrosine-protein kinase Src; mTORC1/2: mechanistic target of rapamycin complex 1/2; TME: tumor microenvironment; CAF: cancer-associated fibroblast; FAK: focal adhesion kinase; ICIs: immune checkpoint inhibitors.

The following sections describe these mechanisms in detail and outline their implications for current and emerging KRAS-targeted therapies.

## INTRINSIC RESISTANCE TO KRAS INHIBITION

An important, underexplored challenge in PDAC is intrinsic resistance to KRAS inhibition. As observed, for example, in lung cancer, a substantial proportion of KRAS-mutant tumors do not derive clinically meaningful benefit from KRAS inhibitors from the outset, despite adequate pharmacological target engagement^[38,39]^. This phenomenon suggests that, in a subset of PDACs, oncogenic KRAS signaling may not be the dominant driver of tumor maintenance, or that parallel survival programs may already be active before therapy initiation. Such baseline resistance contrasts with acquired resistance and reflects pre-existing tumor states that enable survival under KRAS suppression.

Intrinsic resistance may therefore result from several non-mutually exclusive factors. Co-occurring genetic alterations, including loss of tumor suppressor genes such as *TP53*, *CDKN2A*, and *SMAD4*, are frequent in PDAC^[40]^. Although their direct impact on KRAS inhibitor response in PDAC remains incompletely defined, data from lung cancer models indicate that co-mutations can modify therapeutic sensitivity. Hence, these alterations may reshape signaling dependencies and support cell-cycle progression or survival independently of KRAS activity^[41]^. In addition, constitutive activation of RTKs or downstream effectors may maintain MAPK or PI3K pathway output despite pharmacological KRAS inhibition in lung cancer, while similar mechanisms may contribute to pathway activation in KRAS-mutant PDAC^[38,42]^. Transcriptional lineage states, including mesenchymal or stem-like shifts, have also been associated with reduced KRAS dependency and diminished sensitivity to pathway inhibition in colon cancer cell lines^[43]^. In this context, activation of the YAP1 transcriptional coactivator has emerged as a key mediator of KRAS-independent survival, enabling tumor maintenance in the absence of oncogenic KRAS signaling^[43,44]^.

**Figure 3:**
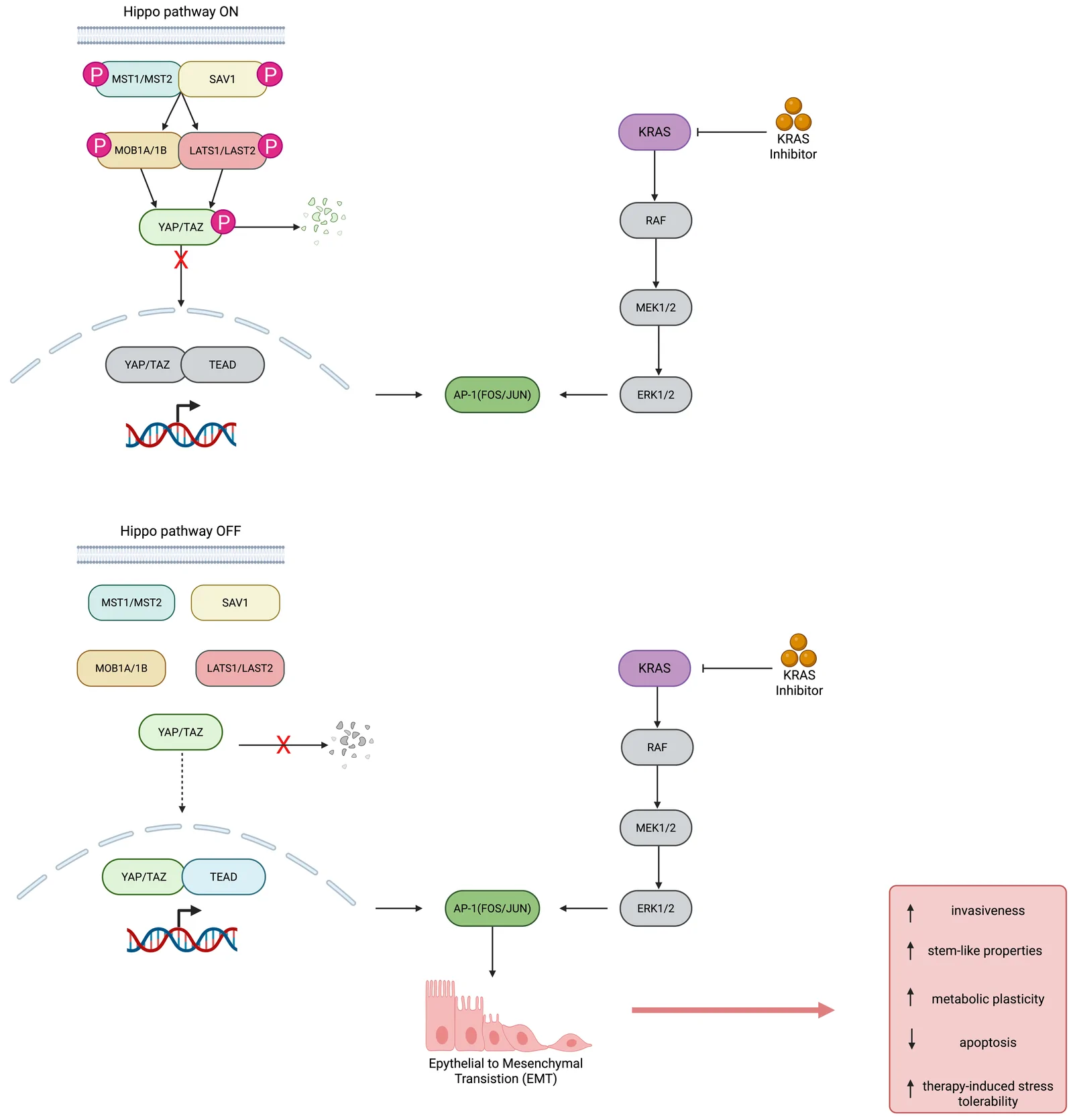
Intrinsic resistance to KRAS inhibition in PDAC driven by Hippo pathway inactivation and AP-1/YAP–TAZ transcriptional cooperation. Pharmacological inhibition of KRAS suppresses MAPK signaling and ERK-dependent AP-1 (FOS/JUN) activity. Inactivation of the Hippo pathway stabilizes and promotes nuclear accumulation of YAP/TAZ, enabling TEAD-dependent transcription and cooperation with AP-1 to sustain KRAS-independent transcriptional programs associated with therapy resistance. Created in BioRender. Giovannetti, E. (2026) https://BioRender.com/69l6sp4. PDAC: Pancreatic ductal adenocarcinoma; AP-1: activator protein 1; YAP: Yes-associated protein; TAZ: transcriptional co-activator with PDZ-binding motif; MAPK: mitogen-activated protein kinase; ERK: extracellular signal-regulated kinase; FOS: Fos proto-oncogene; JUN: Jun proto-oncogene; TEAD: TEA domain transcription factor; RAF: rapidly accelerated fibrosarcoma; MEK1/2: mitogen-activated protein kinase kinase 1/2.

YAP/TAZ activity is tightly regulated by the tumor-suppressive Hippo pathway, a serine/threonine kinase cascade that controls their localization and stability. When Hippo signaling is active, YAP/TAZ are phosphorylated and retained in the cytoplasm or degraded; when inactive, they translocate to the nucleus and interact with transcription factors such as TEAD to drive gene expression programs linked to proliferation, survival, and cellular plasticity. Under these conditions, YAP/TAZ-TEAD complexes cooperate with AP-1 (FOS/JUN), thereby restoring or maintaining transcriptional activity despite KRAS inhibition [Figure 3].

YAP/TAZ are frequently overactive in PDAC, as indicated by increased expression and nuclear localization, and YAP overexpression has been associated with poor patient outcomes. Functionally, YAP hyperactivation can compensate for KRAS loss, sustaining tumor cell viability upon KRAS downregulation, and effectively bypassing KRAS dependency. These preclinical and clinical findings in PDAC support a model in which YAP acts not only downstream of KRAS during tumor initiation but also as a key mediator of KRAS-independent tumor maintenance, thereby contributing to intrinsic resistance to KRAS-targeted therapies^[45,46]^.

Consistent with this model, inactivation of the Hippo pathway increases nuclear YAP activity and is associated with more aggressive disease and metastatic progression, further highlighting the clinical relevance of YAP-driven transcriptional programs in PDAC. Importantly, YAP/TAZ activation is strongly linked to EMT induction, providing a mechanistic connection between Hippo pathway dysregulation and resistance-associated cellular states.

EMT represents a resistance-associated cellular state in PDAC that reduces dependency on KRAS signaling and contributes to intrinsic resistance to KRAS inhibition. This state promotes invasiveness, stem-like properties, and metabolic plasticity, enabling tumor cells to evade apoptosis and tolerate therapy-induced stress. EMT-associated signaling also enhances antioxidant responses and autophagy, supporting the persistence of drug-tolerant cells^[42,45]^. Overall, although the mesenchymal phenotype may also emerge dynamically during treatment and contribute to acquired resistance, the evidence reviewed here predominantly supports its role in pre-existing resistant states. Thus, EMT is considered primarily in the context of intrinsic resistance to KRAS-targeted therapies in PDAC.

Another important resistance mechanism involves “on-target” mutations, which are specific to individual compounds or compound classes that exploit the same binding pocket. These alterations have been extensively studied in both acquired and intrinsic resistance across tumors responding to a wide range of targeted agents^[47]^. This mechanism of intrinsic resistance has also been characterized in KRAS G12C-mutant NSCLC, where a substantial fraction of patients exhibits minimal or no clinical response despite evidence of on-target drug engagement^[10,39,48]^.

In contrast, in PDAC, the extent and molecular basis of intrinsic resistance remain poorly defined. This is largely due to limited clinical experience with KRAS inhibitors and the lack of systematic studies interrogating baseline resistance states, representing a critical unmet need in the field. In summary, most proposed mechanisms of intrinsic resistance to KRAS inhibitors in PDAC, including co-occurring genetic alterations and constitutive RTK activation, are largely inferred from evidence in NSCLC and CRC and therefore remain biologically plausible rather than directly validated in pancreatic cancer. In contrast, the Hippo-YAP/TAZ axis is more strongly supported in PDAC, while EMT represents an additional indirect indicator of a resistant cellular state. Finally, on-target mutations constitute an anticipated resistance mechanism common to targeted therapies.

## KRAS-DEPENDENT MECHANISMS

The transition of KRAS from an undruggable oncogene to a clinically targetable one has provided a therapeutic opportunity to study this oncogene-centered resistance. The introduction of covalent inhibitors targeting the GDP-bound KRAS G12C conformation has demonstrated that direct KRAS inhibition can achieve meaningful clinical responses, particularly in NSCLC, as shown in trials such as CodeBreaK 100 (NCT03600883) and KRYSTAL-1 (NCT03785249). However, the limited durability of responses and modest efficacy observed in PDAC highlight the rapid emergence of resistance mechanisms that remain fundamentally dependent on KRAS itself.

One of the best-described on-target resistance mechanisms is the emergence of secondary mutations within KRAS, particularly in tumors treated with covalent inhibitors. Evidence from both preclinical studies, including engineered Ba/F3 cellular models, and clinical studies in patients with NSCLC and CRC has shown that mutations adjacent to the switch pocket II, such as Y96C, H95D/Q/R, R68, or Q66L, directly impair engagement of KRAS G12C inhibitors such as sotorasib, adagrasib, and related covalent compounds, and thereby reduce binding affinity or alter the dynamic requirements for covalent engagement^[15,49,50]^. These mutations have been documented across KRAS inhibitor-treated tumors and can restore downstream MAPK signaling despite continued drug exposure^[50]^. In PDAC, where G12D and G12V mutations predominate, secondary mutations may similarly emerge under selective pressure from allele-specific inhibitors such as MRTX1133, although clinical evidence remains limited.

Another mechanism involves *KRAS* amplification, in which tumor cells increase mutant KRAS levels to overcome pharmacological inhibition. Rather than acquiring new mutations, resistant clones can upregulate KRAS through gene amplification or by shifting allelic expression in favor of the mutant variant, resulting in substantially higher KRAS protein levels. This increase saturates the available inhibitor molecules, allowing a portion of KRAS proteins to remain active and maintain downstream MAPK signaling^[15,51]^.

Evidence for this mechanism has been reported across multiple tumor types. In CRC models, *KRAS* gene amplification has been identified as a dominant resistance mechanism following KRAS G12C inhibition, restoring ERK activity and promoting sustained proliferation^[51]^.

Importantly, similar mechanisms have been identified in preclinical models of NSCLC, CRC, and PDAC, where KRAS copy-number gain and enrichment of the mutant allele have been shown to mediate escape from vertical pathway suppression, reinforcing the dependency of resistant cells on amplified KRAS signaling^[15,18,52]^. Consistent with these findings, additional preclinical studies indicate that KRAS amplification can emerge following treatment with KRAS inhibitors such as sotorasib, adagrasib, and the KRAS G12D inhibitor MRTX1133, supporting its role as a potential on-target resistance mechanism capable of restoring KRAS pathway output in PDAC^[18]^.

Many KRAS inhibitors bind preferentially to the GDP-loaded conformation; thus, resistance can arise when tumor cells accelerate nucleotide exchange or diminish GTPase activity. Enhanced signaling through upstream RTKs promotes activation of SOS1 and SHP2, two critical regulators of GDP-GTP cycling. Hofmann *et al*. demonstrated that increased SOS1-mediated nucleotide exchange restores RAS-GTP levels in the pancreatic cancer cell line MIA PaCa-2 and undermines KRAS inhibition, while pharmacological SOS1 blockade enhances inhibitor efficacy^[53]^. Similarly, Fedele *et al.* showed in both pancreatic and lung cancer cell lines that SHP2 is required to sustain KRAS-GTP formation downstream of RTKs, and that SHP2 inhibition significantly attenuates KRAS-driven signaling^[54]^.

Loss of GAP function constitutes another route to increased GTP loading. In particular, Stites *et al.* showed, using *in vitro* and *in vivo* models of NSCLC and PDAC, that impaired GAP activity shifts the RAS equilibrium toward persistent GTP binding, reinforcing pathway activation^[55]^. Through these combined mechanisms, including hyperactivation of GEFs and suppression of GAPs, tumor cells can maintain KRAS in a drug-refractory state, thus enabling continued MAPK signaling despite pharmacological KRAS blockade. These KRAS-dependent resistance mechanisms have important therapeutic implications and have directly informed the development of next-generation KRAS-targeted strategies. Multiple KRAS G12C inhibitors with distinct binding properties are currently under clinical evaluation, including divarasib (NCT04449874), olomarasib (NCT04956640), RMC-6291 (NCT05462717), and JDQ443 (NCT04699188), to overcome resistance associated with altered binding affinity or KRAS reactivation [Table 1].

Early-phase studies of divarasib and olomarasib have reported partial responses and disease stabilization but also highlight the persistence of resistance despite improved pharmacological properties in different solid tumor types, including PDAC^[56,57]^. Among allele-specific KRAS G12D inhibitors, MRTX1133 has shown marked tumor regression in preclinical PDAC models. Nevertheless, resistant tumors eventually emerged and displayed substantial molecular heterogeneity. Notably, KRAS amplification was detected in two resistant cases, supporting increased mutant KRAS copy number as a plausible on-target resistance mechanism capable of restoring KRAS pathway output despite continued inhibitor exposure^[18]^. Ongoing clinical studies targeting KRAS G12D, including HRS-4642 (NCT05533463), ASP3082 (NCT05382559), and RMC-9805 (NCT06040541), will be critical to determine whether KRAS-dependent escape mechanisms, including secondary KRAS alterations, KRAS amplification, and altered nucleotide-state regulation, translate to the clinical setting.

Collectively, the persistence of resistance mechanisms centered on KRAS itself has prompted the development of broader RAS-targeting strategies aimed at limiting compensatory signaling. Agents such as RMC-6236/daraxonrasib, currently under clinical evaluation (NCT05379985, NCT06625320, NCT07252232), target active RAS conformations across multiple alleles and have demonstrated antitumor activity in KRAS-mutant PDAC.

In a phase 1/2 study (NCT05379985), initial signals of activity and manageable safety were observed in previously treated patients, supporting subsequent evaluation in the first-line setting^[58]^. Early data from patients in the first-line setting indicate an ORR (~50%) together with evidence of on-target pathway suppression, as reflected by marked reductions in ctDNA RAS variant allele frequency and molecular clearance in a substantial proportion of patients. Toxicity was overall manageable, with predominantly low-grade treatment-related adverse events and limited high-grade toxicity^[58]^.

Importantly, clinical evidence for daraxonrasib has substantially strengthened with the results of the phase III RASolute 302 trial presented at the 2026 ASCO Annual Meeting. In previously treated metastatic PDAC, daraxonrasib significantly improved overall and PFS compared with chemotherapy, with median OS in the G12 population of 13.2 *vs.* 6.7 months and median PFS of 7.2 *vs.* 3.6 months, while grade ≥ 3 adverse events occurred in 61.8% and 69.6%, respectively. These results provide the first phase III evidence that multi-selective RAS(ON) inhibition can produce clinically meaningful benefit in PDAC and compare favorably with the more modest activity previously reported with allele-specific KRAS inhibitors in this disease^[59]^. In comparison, the previously mentioned phase II studies of sotorasib and adagrasib in KRAS G12C-mutant PDAC reported ORRs of approximately 21% and 33%, with median PFS of 4.0 and 5.4 months, respectively, while early clinical studies of KRAS G12D-directed agents have generally remained at an earlier stage of development^[12,23]^.

These findings have led to the initiation of a global randomized phase III trial evaluating RMC-6236 (daraxonrasib) with or without chemotherapy in the first-line metastatic setting (RASolute 303 - NCT07491445). Overall, these approaches will help determine whether oncogene-centered adaptation can be effectively suppressed or whether KRAS-driven resistance remains resilient even to pan-RAS inhibition. These major KRAS-dependent resistance mechanisms identified to date, including secondary KRAS mutations, KRAS amplification, and alterations in nucleotide-state regulation, are supported by both preclinical and clinical evidence across multiple KRAS-driven malignancies. In PDAC, initial *in vitro* and *in vivo* preclinical studies suggest that KRAS reactivation may similarly represent a relevant mechanism of therapeutic escape. However, the relative contribution, frequency, and clinical relevance of these mechanisms in patients with PDAC remain to be established, highlighting a key area for future clinical investigation.

## KRAS-INDEPENDENT MECHANISMS

KRAS-independent bypass signaling encompasses resistance mechanisms through which PDAC cells restore downstream proliferative output via alternative upstream inputs, despite effective pharmacological suppression of mutant KRAS. Owing to the extensive signaling redundancy and strong RTK-RAS coupling in PDAC, activation of RTKs can rapidly reconstitute MAPK and PI3K–AKT–mTOR pathway activity, enabling continued tumor growth independently of the inhibited KRAS allele. Prominent RTKs involved in this process include EGFR, ERBB2, FGFR1, IGF1R, and MET^[60-65]^.

Direct evidence for this adaptive rewiring in PDAC comes from studies interrogating KRAS suppression in pancreatic models. In a shRNA-based model of endogenous KRAS suppression in murine PDAC, Chen *et al*. found that the majority of PDAC cells tolerated acute and sustained KRAS silencing by entering an adapted state with preserved viability and capacity to re-expand once KRAS expression was restored, consistent with robust bypass signaling and network rewiring^[64]^. Moreover, Ryan *et al*. reported that RTK-driven bypass is not a rare event but rather a dominant adaptation^[60]^; indeed, most KRAS-mutant models examined, including CRC, NSCLC, and PDAC models, show rapid recovery of ERK signaling through alternative RTK inputs once the KRAS or MEK node is suppressed^[60]^.

Mechanistically, this adaptive rewiring is largely driven by activation of upstream RTKs following KRAS inhibition. Increased RTK activity restores downstream MAPK and PI3K signaling, enabling tumor cells to maintain proliferative output despite suppression of mutant KRAS. In PDAC models treated with KRAS G12D inhibitors, this response has been linked to upregulation and activation of ERBB family receptors, resulting in partial recovery of downstream signaling pathways^[62]^.

These observations are consistent with earlier work by Lito *et al.*^[61]^, which, although conducted in a BRAF V600E-driven model rather than KRAS-mutant PDAC, established a conceptual framework by demonstrating that MAPK pathway inhibition can relieve ERK-dependent negative feedback on RTKs, thereby promoting ligand-driven signaling and pathway rebound^[62-65]^.

Moreover, in several PDAC models, RTK-driven reactivation may engage wild-type RAS paralogues, including NRAS and HRAS, which share common downstream effectors such as RAF and PI3K, thereby providing an alternative route to sustain signaling^[66]^.

Mechanistic support for this paradigm comes from studies in other oncogenic contexts, where wild-type RAS has been shown to contribute to tumor cell fitness and mediate resistance to MAPK pathway inhibition. In particular, Ambrogio *et al.* demonstrated in lung cancer models that wild-type RAS contributes to resistance to MEK inhibition, in part through RAS dimerization and maintenance of downstream ERK signaling^[63]^. While derived from lung adenocarcinoma, these findings support the broader concept of RTK–RAS network plasticity, which may also be relevant in PDAC as a potential bypass mechanism to KRAS inhibition.

Preclinical studies in KRAS-driven lung cancer also provide convincing mechanistic support for RTK-mediated bypass signaling as a resistance mechanism to KRAS and MAPK pathway inhibition. Although outside the context of PDAC, these findings are highly relevant for understanding conserved adaptive resistance mechanisms across KRAS-mutant tumors. For instance, early work by Turke *et al.* demonstrated that MEK inhibition in EGFR- and HER2-driven cancer cells, primarily from lung and breast cancer models, induces rapid compensatory activation of the PI3K-AKT pathway through ERBB3 hyperactivation, driven by relief of inhibitory phosphorylation within ERBB family receptors^[65]^. This feedback mechanism restored downstream survival signaling despite effective MAPK pathway suppression, illustrating how RTK signaling can dynamically rewire intracellular networks under targeted therapy pressure.

More recently, studies in KRAS G12C-mutant NSCLC have directly linked RTK amplification to resistance against KRAS inhibitors. Suzuki *et al*. showed that acquired resistance to sotorasib was driven by subclonal MET amplification, which restored MAPK signaling through enhanced RAS-GTP cycling while simultaneously activating AKT in a RAS-independent manner^[67]^. Pharmacological MET inhibition resensitized resistant cells and xenografts to KRAS inhibition, highlighting MET as a functional bypass node. Similarly, Misale *et al.* demonstrated heterogeneous intrinsic responses to KRAS G12C inhibition across NSCLC models, driven by adaptive reactivation of MAPK signaling and incomplete suppression of the PI3K-AKT pathway^[39]^. This work also showed that co-targeting PI3K–AKT–mTOR signaling restored sensitivity to KRAS inhibition in resistant models, including patient-derived xenografts.

Remarkably, clinical data in PDAC further support the biological importance of RTK-driven signaling. In a cohort of patients with resected PDAC (*n* = 131), Wu *et al.* evaluated EGFR and C-X-C motif chemokine receptor 4 (CXCR4) expression and found that co-expression of these markers identified a subset of patients with more aggressive clinicopathological features, including higher rates of lymph node metastasis and perineural invasion, as well as shorter disease-free survival (DFS) (*P* < 0.001) and OS (*P* = 0.001) compared with patients negative for one or both markers^[68]^. Although this study did not involve KRAS inhibitors, it provides evidence that enhanced EGFR-axis activity is clinically associated with more aggressive disease biology in PDAC, consistent with its proposed role as a bypass driver in experimental models.

Similar patterns are observed for the RTK c-MET. Neuzillet *et al*. analyzed 149 patients with stage I-II PDAC and developed a robust immunostaining score for c-MET; high c-MET expression was independently associated with shorter DFS [hazard ratio (HR) = 3.5, *P* < 0.001] and OS (HR = 4.3, *P* < 0.001)^[69]^. A subsequent meta-analysis by Kim *et al*.^[70]^, including 423 surgically resected PDAC patients from five studies, confirmed that c-MET overexpression is associated with worse OS (pooled HR = 2.0 for high *vs.* low c-MET expression). Collectively, these clinical data show that RTKs commonly implicated as bypass drivers, such as EGFR and c-MET, are associated with inferior survival in PDAC, consistent with a potential role within adaptive signaling networks in the context of disrupted KRAS signaling.

An additional KRAS-independent resistance mechanism may involve drug efflux mediated by adenosine triphosphate (ATP)-binding cassette transporters, particularly ABCB1 (P-glycoprotein), which actively exports anticancer drugs and reduces their intracellular accumulation. Recent PDAC-specific preclinical evidence of this mechanism was provided by Dilly *et al.*, who demonstrated that KRAS-driven PDAC cells can exploit ABCB1-mediated efflux to limit exposure to RAS-targeted therapies^[18]^. In this study, ABCB1 activity constrained effective drug accumulation in tumor cells, attenuating pathway suppression without requiring reactivation of KRAS signaling itself, thereby supporting an established role for transporter-mediated resistance in PDAC. Mechanistic support for this concept is provided by studies in other tumor settings. A biophysical analysis has shown that ABCB1 overexpression is sufficient to reduce intracellular concentrations of structurally diverse small-molecule kinase inhibitors, thereby conferring a multidrug-resistant phenotype independent of target alteration^[71]^. Furthermore, in a pharmacokinetic setting, Rijmers *et al*. demonstrated that ABCB1 can measurably restrict brain penetration of a KRAS inhibitor, reinforcing the broader point that transporter activity can shape effective drug exposure *in vivo*, even when the molecular target remains druggable^[72]^.

Proto-oncogene tyrosine-protein kinase Src (SRC) has emerged in recent years as a key mediator of adaptive and acquired resistance mechanisms across multiple tumor types. SRC is a membrane-associated non-RTK that overlaps functionally with KRAS signaling, as it can activate both the RAF–MEK–ERK and PI3K–AKT pathways and modulate transcriptional regulators such as signal transducer and activator of transcription 3 (STAT3) and β-catenin^[73]^. Through these activities, SRC influences diverse tumor phenotypes, including cell survival, motility, metabolic adaptation, and therapy resistance. A study combining preclinical and clinical evidence from several KRAS-mutant malignancies, including PDAC, showed that SRC is hyperactivated after the pharmacological inhibition of RAS signaling, indicating that SRC is associated with pathway rewiring and therapeutic escape^[73]^. Mechanistically, SRC has been linked to the reactivation of downstream MAPK signaling through ERK and JNK, promotion of cell-cycle progression via upregulation of cyclin D1, and suppression of regulated cell-death programs. In addition, SRC drives transcriptional programs; it can modulate the expression of drug-efflux transporters such as ABCB1, potentially reducing intracellular drug accumulation. Collectively, these findings position SRC as a central signaling hub that sustains tumor viability and limits the durability of RAS-targeted therapies^[73]^. Several KRAS-independent bypass mechanisms have been defined mainly outside PDAC, including wild-type RAS paralogue engagement, ERBB3-mediated feedback activation, MET amplification, and incomplete suppression or adaptive reactivation of MAPK and PI3K-AKT signaling, mostly in NSCLC, CRC, breast cancer, or other oncogene-dependent models. In PDAC, these mechanisms remain largely biologically plausible but indirectly supported; for example, the clinical association of EGFR and c-MET expression with aggressive disease highlights the biological relevance of RTK signaling in this context. More directly relevant evidence in PDAC supports reversible adaptive states after KRAS suppression, RTK-mediated bypass signaling, ABCB1-mediated drug efflux, and SRC-associated signaling rewiring, mainly in preclinical models. However, evidence varies across these mechanisms, with ABCB1-mediated efflux and SRC rewiring supported by a limited number of studies. Overall, the clinical relevance of these bypass routes in PDAC remains to be defined through dedicated clinical studies.

## DOWNSTREAM PATHWAY REACTIVATION

Even when KRAS itself or its immediate upstream activators are effectively inhibited, PDAC cells can re-establish oncogenic signaling through downstream pathway reactivation. Direct experimental evidence for this phenomenon in PDAC comes primarily from Brown *et al.*, who systematically examined the consequences of genetic and pharmacological KRAS suppression in human and mouse pancreatic cancer models^[74]^. They demonstrated that acute KRAS or MEK inhibition leads to rapid adaptive reactivation of the MAPK pathway, driven by increased activity of multiple upstream RTKs and engagement of wild-type RAS isoforms. Importantly, ERK signaling was restored despite sustained KRAS suppression, establishing downstream pathway reactivation as a resistance mechanism in KRAS-inhibited PDAC rather than a theoretical extension from other tumor types^[74]^.

Beyond this study, however, there is a scarcity of work directly interrogating downstream pathway reactivation in PDAC tumors treated with KRAS inhibitors. As a result, much of the current understanding must be inferred from mechanistic studies in related oncogenic contexts and from PDAC studies that target downstream pathways independently of KRAS inhibition. This gap in the literature represents a major limitation in defining resistance mechanisms specific to KRAS-targeted therapies in pancreatic cancer.

The recognition of downstream pathway reactivation as a key resistance mechanism to KRAS inhibition provides a strong rationale for therapeutic strategies aimed at targeting critical downstream effectors, including the MAPK and PI3K–AKT–mTOR pathways^[39,74]^.

Efforts to inhibit the PI3K–AKT–mTOR branch of RAS signaling have produced mixed outcomes in PDAC, despite its clear biological relevance. Rigosertib, a multikinase inhibitor that promotes apoptosis through PI3K/AKT pathway suppression, failed to improve outcomes when added to gemcitabine in a phase II/III study of previously untreated PDAC^[75]^. While this study did not involve KRAS inhibition, it illustrates the intrinsic capacity of PDAC cells to tolerate suppression of major downstream survival pathways. Nevertheless, the pathway remains of therapeutic interest, especially in the context of resistance to KRAS-targeted therapies. Ongoing clinical evaluation of the KRAS G12C inhibitor divarasib (NCT04449874) includes combination strategies with the PI3K inhibitor inavolisib, reflecting a recognition that co-targeting PI3K signaling may help overcome resistance to KRAS inhibition in advanced KRAS G12C PDAC.

A parallel attempt to target the MAPK cascade revealed a different set of challenges. Although ERK1/2 inhibitors have entered clinical evaluation, monotherapy has shown limited promise. In the HERKULES-1 I/IIb study, most PDAC patients treated with the ERK inhibitor ERAS-007 discontinued therapy early due to rapid disease progression^[76]^. These modest responses mirror earlier findings with MEK inhibitors, where selumetinib and trametinib failed to extend OS in combination with gemcitabine in PDAC patients^[77,78]^. Although these studies predate the development of KRAS inhibitors, they provide important context: suppression of MAPK signaling alone is insufficient in PDAC and is prone to rapid adaptive escape, a principle that is directly relevant to KRAS-targeted therapies.

A promising therapeutic strategy emerged from the recognition that MEK inhibition triggers feedback loops. In KRAS-mutant models, MEK inhibition paradoxically promotes the formation of RAF-MEK complexes through feedback signaling loops, partially restoring MAPK pathway activity^[61]^. This feedback loop is highly relevant in the setting of KRAS inhibition, where partial downstream suppression may similarly trigger compensatory signaling. Sotorasib (NCT05074810) and adagrasib (NCT05375994), both in combination with avutometinib (VS-6766), a dual RAF/MEK inhibitor engineered to block this feedback reactivation, are currently under a phase I evaluation in patients who previously received KRAS inhibitors, as well as in combination regimens with chemotherapy and the focal adhesion kinase (FAK) inhibitor defactinib in PDAC. Of note, early clinical signals suggest encouraging activity^[79]^.

Additional preclinical work has revealed therapeutic vulnerabilities that emerge when MEK inhibition is paired with autophagy-targeting therapies. Autophagy has emerged as a critical adaptive survival mechanism in PDAC, particularly under therapeutic stress. As a highly conserved catabolic process, autophagy enables tumor cells to recycle intracellular components and sustain energy production and biosynthesis under conditions of nutrient deprivation^[80]^. Key metabolic sensors, including AMPK and mTOR, tightly regulate this process and converge on the ULK1 complex to initiate autophagosome formation. Notably, oncogenic KRAS signaling promotes autophagy through coordinated regulation of AMPK and mTORC1, with AMPK promoting ULK1 activation and mTORC1 exerting an inhibitory effect on ULK1-dependent autophagy, thereby supporting metabolic homeostasis and tumor growth. In addition, MYC stabilization downstream of KRAS–ERK signaling enhances transcriptional programs involved in autophagy and lysosomal function, further linking metabolic demand to intracellular recycling. In this context, inhibition of MAPK signaling may increase tumor dependency on autophagy, providing a strong rationale for combined targeting of these pathways^[11,80,81]^.

**Figure 4:**
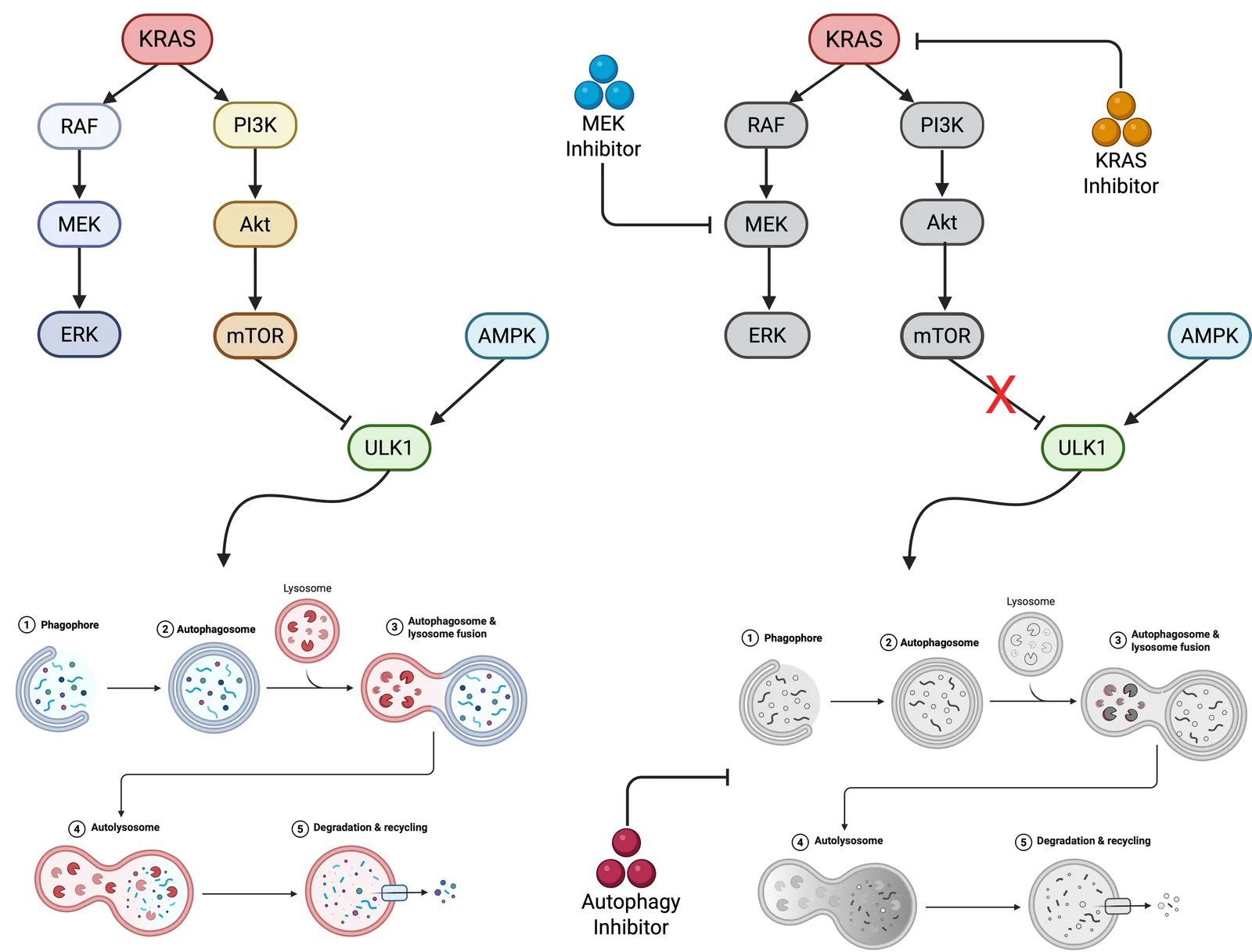
KRAS-regulated signaling networks controlling autophagy and adaptive responses in PDAC. Under basal conditions, oncogenic KRAS coordinates autophagy regulation through two major downstream pathways: the PI3K–AKT–mTOR axis and the MAPK cascade. Through PI3K–AKT signaling, KRAS promotes mTORC1 activation, which suppresses autophagy initiation by inhibiting ULK1. In parallel, AMPK positively regulates ULK1, promoting autophagy induction under metabolic stress, while MAPK signaling supports tumor growth and metabolic adaptation. Upon pharmacological KRAS inhibition, both MAPK and PI3K–AKT–mTOR signaling are suppressed (greyed out), leading to adaptive rewiring of the system. In this context, loss of mTOR-mediated repression permits sustained ULK1 activation driven by persistent AMPK signaling. This creates strong pro-autophagic pressure, a key adaptive survival mechanism in PDAC. Concurrent MEK inhibition further reinforces MAPK pathway suppression. However, when autophagy is simultaneously inhibited, the autophagic process is blocked downstream despite continued ULK1 activation, thereby eliminating a critical metabolic escape route and promoting tumor cell vulnerability. This schematic illustrates a proposed mechanistic model integrating current preclinical evidence on adaptive signaling and autophagy following KRAS inhibition in PDAC. Created in BioRender. Giovannetti, E. (2026) https://BioRender.com/uu2e1sj. PDAC: Pancreatic ductal adenocarcinoma; PI3K: phosphoinositide 3-kinase; AKT: AKT serine/threonine kinase; mTOR: mechanistic target of rapamycin; MAPK: mitogen-activated protein kinase; mTORC1: mechanistic target of rapamycin complex 1; ULK1: Unc-51 like autophagy activating kinase 1; AMPK: AMP-activated protein kinase; MEK: mitogen-activated protein kinase kinase; RAF: rapidly accelerated fibrosarcoma; ERK: extracellular signal-regulated kinase.

Consistently, studies using PDAC cell lines and patient-derived xenografts have demonstrated that MEK inhibition disrupts autophagy regulation, and that co-targeting MEK and autophagy can produce strong synergistic suppression of tumor growth [Figure 4]^[82]^. Although hydroxychloroquine has been widely explored as an autophagy inhibitor in PDAC, its mechanism of action through lysosomal inhibition lacks specificity, potentially affecting other nutrient-scavenging processes such as micropinocytosis^[80]^. Nonetheless, early-phase clinical studies are actively evaluating autophagy-targeting strategies in combination with MAPK pathway inhibitors, including trametinib (NCT03825289), binimetinib (NCT04132505), ulixertinib (NCT04145297), and LY3214996 (NCT04386057).

Beyond autophagy and nucleotide metabolism, metabolic adaptations involving glucose and glutamine utilization may also contribute to the persistence of KRAS-driven PDAC under therapeutic stress. Oncogenic KRAS promotes glucose uptake and glycolytic flux through increased expression of GLUT1 and glycolytic enzymes, including hexokinases, phosphofructokinase-1, and lactate dehydrogenase A, thereby supporting ATP production and biosynthetic activity. In parallel, PDAC cells exhibit a pronounced dependence on glutamine to replenish tricarboxylic acid (TCA) cycle intermediates and sustain anabolic and redox metabolism. KRAS-driven glutamine utilization is mediated, in part, through a non-canonical pathway involving increased glutamic-oxaloacetic transaminase 1 (GOT1) activity, which supports NADPH production and maintenance of cellular redox homeostasis^[80,81]^.

These metabolic dependencies may represent therapeutically exploitable vulnerabilities when combined with pathway-directed treatments. Although pharmacological inhibition of glutaminase (GLS) alone has shown limited antitumor activity in preclinical PDAC models, broader inhibition of glutamine metabolism with the glutamine antagonist 6-diazo-5-oxo-L-norleucine (DON) suppresses PDAC growth and metastasis. Importantly, DON treatment induces an adaptive increase in asparagine synthetase (ASNS), and co-targeting this response with L-asparaginase enhances DON-mediated suppression of PDAC cell fitness and reduces metastatic progression in preclinical models. Notably, glutamine/asparagine metabolism can also intersect with MAPK signaling: asparagine depletion enhances ERK1/2 phosphorylation, while combined asparaginase and MEK inhibition produces greater suppression of orthotopic PDAC tumor growth than either treatment alone^[83]^. These findings support the concept that metabolic adaptations can both sustain tumor fitness and activate compensatory signaling responses, providing a rationale for combining metabolic interventions with inhibition of KRAS downstream effectors^[83]^.

Lipid metabolism represents another metabolic axis rewired by oncogenic KRAS in PDAC. In addition to promoting glycolytic and amino acid metabolism, KRAS regulates lipid storage and utilization through the MAPK/ERK-dependent suppression of hormone-sensitive lipase (HSL), resulting in accumulation of lipid droplets within tumor cells. These stored lipids can subsequently be mobilized during invasive migration, increasing fatty-acid oxidation and oxidative metabolism to support the energetic demands of metastatic dissemination. Conversely, restoration of HSL expression depletes lipid stores, shifts cells toward oxidative metabolism, and suppresses tumor progression and metastasis in preclinical models^[84]^. Inhibition of lipid utilization, including mitochondrial fatty-acid transport, further impairs ATP production and invasive migration, suggesting that KRAS-driven lipid storage and utilization may represent an additional metabolic vulnerability that could be exploited therapeutically^[84]^.

Importantly, autophagy is not the only metabolic adaptation engaged under therapeutic stress. KRAS-inhibitor-resistant PDAC cells can preserve nucleotide biosynthesis despite pathway suppression by maintaining MYC-RPIA-dependent flux through the non-oxidative pentose phosphate pathway. This allows survival even in the absence of full MAPK pathway activity, representing downstream functional reactivation at the metabolic level^[85]^. Notably, MEK inhibition largely recapitulates these metabolic effects but remains predominantly cytostatic, further highlighting the need for combination strategies targeting both signaling pathways and downstream metabolic dependencies^[85]^.

Collectively, these available studies illustrate that downstream RAS effector pathways, both MAPK and PI3K–AKT–mTOR, remain highly adaptive and can be rapidly reactivated when inhibited along a single axis. However, aside from the work of Brown *et al.*, direct studies of downstream reactivation in KRAS-inhibited PDAC remain limited^[74]^. Bridging this gap will require systematic investigation of pathway dynamics in tumors treated with RAS inhibitors, as well as rational combination strategies designed to suppress feedback reactivation and adaptive survival programs^[74]^. Available evidence therefore supports downstream reactivation of the MAPK and PI3K–AKT–mTOR pathways as a biologically relevant mechanism in PDAC. Direct evidence comes from preclinical models showing adaptive recovery of ERK signaling after KRAS suppression, whereas clinical data on PI3K, MEK, and ERK inhibitors, obtained in non-KRAS-targeted settings, indirectly support this phenomenon. In addition, autophagy and metabolic adaptations, including MYC-RPIA-dependent reliance on the non-oxidative pentose phosphate pathway, represent emerging vulnerabilities supported predominantly by preclinical evidence. Overall, these findings highlight a major translational gap between mechanistic evidence and clinical validation in KRAS inhibitor-treated PDAC.

## TME-MEDIATED RESISTANCE

In PDAC, mutant KRAS contributes not only to tumor cell-intrinsic signaling but also to establishing a highly desmoplastic, stroma-rich microenvironment, thereby playing a dominant role in shaping the tumor ecosystem. This feature is particularly relevant in pancreatic cancer and differs in several respects from other KRAS-mutant tumor contexts. The contribution of the TME to KRAS inhibitor response in PDAC should therefore be interpreted within the specific pancreatic context. Accordingly, the evidence discussed below focuses exclusively on pancreatic cancer studies, including preclinical and clinical data that directly or indirectly support a role for stroma and TME-mediated resistance to KRAS-directed therapies^[86]^.

Clinical efforts to therapeutically modulate the dense stroma in PDAC have so far yielded limited benefit, highlighting the complexity of stromal-mediated resistance to KRAS-directed and other systemic therapies. Early strategies aimed at broadly disrupting the stromal architecture, including inhibition of matrix metalloproteinases or blockade of Sonic Hedgehog signaling, failed to improve clinical outcomes in advanced PDAC and, in some cases, were associated with adverse effects on disease progression^[87-89]^. Similarly, enzymatic depletion of hyaluronan using pegylated recombinant human hyaluronidase (PEGH20) improved PFS (6.0 *vs.* 5.3 months) in combination with gemcitabine/nab-paclitaxel in a phase II trial^[90]^, but this benefit did not translate into improved OS in another phase II trial combined with FOLFIRINOX in patients with metastatic PDAC^[91]^. A subsequent phase III trial confirmed that combining PEGH20 with gemcitabine/nab-paclitaxel did not improve OS compared with chemotherapy alone (median OS 11.2 *vs.* 11.5 months)^[92]^.

The limited efficacy of these approaches can be attributed to the distinctive biological and structural characteristics of the PDAC stroma. The desmoplastic microenvironment is defined by extensive deposition of collagen-rich extracellular matrix and hyaluronan, resulting in elevated interstitial fluid pressure, vascular compression, and impaired intratumoral perfusion. These physicochemical constraints limit drug penetration and create heterogeneity in intratumoral drug exposure, thereby reducing the effectiveness of small-molecule inhibitors, including KRAS-targeted agents^[93,94]^.

Beyond these physical barriers, the TME actively contributes to therapeutic resistance through complex cellular and molecular interactions. It is characterized by a highly immunosuppressive milieu enriched in macrophages, myeloid-derived suppressor cells, dendritic cells, and neutrophils, which collectively promote tumor progression, chemoresistance, and immune evasion. Notably, oncogenic mutant KRAS signaling plays a central role in shaping this immunosuppressive landscape by promoting myeloid cell recruitment while limiting infiltration and function of cytotoxic T cells and antigen-presenting cells^[95]^.

In parallel, the stromal compartment in PDAC exhibits marked cellular heterogeneity. Transcriptomic analyses identified multiple cancer-associated fibroblast (CAF) subpopulations, including myofibroblast, inflammatory, and antigen-presenting phenotypes, each associated with distinct signaling programs that support tumor progression and modulate therapeutic response^[96-98]^. Consistent with this complexity, therapeutic responses to KRAS inhibition are increasingly recognized as being shaped by reciprocal interactions between tumor cells and the surrounding stroma.

Beyond their structural and immunomodulatory functions, CAFs also directly attenuate the efficacy of KRAS-directed therapies through paracrine signaling. CAF-derived growth factors, including FGF1, HBEGF, and TGFβ family ligands, reinforce RTK signaling in tumor cells, thereby sustaining downstream proliferative signaling and limiting the durability of KRAS pathway inhibition.

In particular, CAF-derived FGF1 activates FGFR signaling and sustains MYC stability through the AKT/GSK3β axis, preventing complete MYC suppression following RAS/MAPK pathway inhibition. Notably, pharmacological reprogramming of CAFs through FAK inhibition reduced FGF1 production, restored MYC suppression, and reversed stromal-mediated resistance in preclinical models, highlighting that adaptive resistance arises not only from tumor cell-intrinsic mechanisms but also from reciprocal tumor–stroma interactions^[99]^.

In immunocompetent PDAC models, inhibition of KRAS signaling, such as with KRAS G12C/G12D inhibitors or the pan-RAS(ON) inhibitor RMC-7977, has been shown to induce a more inflamed microenvironment, characterized by increased infiltration of cytotoxic CD8+ T cells, expansion of CD4+ T helper cells, enrichment of B cell subsets, a higher M1/M2 macrophage ratio, and reduced abundance of granulocytic and myeloid suppressor populations. In parallel, these interventions are associated with enhanced antitumor immunity and synergistic responses when combined with immune checkpoint blockade, highlighting the importance of sustained CD8+ T-cell activity for durable tumor control^[95,100]^.

Similarly, BI-2493, a first-in-class pan-KRAS inhibitor with activity against multiple KRAS alleles, has demonstrated broad antitumor activity across PDAC cell lines and patient-derived xenografts. Treatment with BI-2493 significantly remodels the TME, including increased intratumoral CD8+ effector T cells, reduced infiltration of myeloid populations, and a shift toward a more immunostimulatory environment that enables increased sensitivity to immune checkpoint inhibitors^[95]^. Notably, early in treatment, tumors display enhanced infiltration of CD4+ T helper cells, CD8+ cytotoxic T cells, and specific B cell subsets that may support T cell expansion and activation. In addition, reductions in eosinophils and immunosuppressive tumor-associated macrophages, along with decreased exhaustion of CD8+ T cells, further underscore the immunomodulatory effects of KRAS pathway inhibition beyond direct tumor cell targeting^[95]^.

However, despite these initial immunostimulatory effects, prolonged treatment with BI-2493 leads to therapeutic resistance and eventual tumor progression^[95]^. This acquired resistance is associated with increased YAP1 signaling within tumor cells and a concurrent reprogramming of the TME toward a more immunosuppressive state, characterized by reduced relative abundance of T and B cells and increased infiltration of M2-polarized macrophages and granulocytes. In parallel, upregulation of immune checkpoint molecules within the TME contributes to impaired T cell effector function. These observations suggest that adaptive resistance mechanisms involve coordinated tumor cell–intrinsic (YAP-driven) and microenvironmental remodeling that collectively restore immune evasion^[43,95]^.

Overall, these findings highlight the TME as a dynamic and bidirectional regulator of therapeutic response in PDAC. While KRAS inhibition can transiently reprogram the microenvironment toward a pro-inflammatory state and sensitize tumors to immune checkpoint blockade, resistance is associated with a reversal toward immunosuppression. This defines a potential therapeutic window in which early combination strategies integrating KRAS pathway inhibition and immune checkpoint blockade may maximize durable antitumor immunity before the emergence of adaptive resistance programs. Such strategies should focus on combining KRAS inhibitors with immunotherapy agents that can promote T-cell infiltration in early stages of PDAC development, to reverse immunosuppression and ultimately turn “cold” (T-cell low) PDAC tumors “hot” (T-cell high).

In line with the dynamic and adaptive nature of the PDAC TME, similar principles of stromal plasticity and compensatory resistance are observed in therapeutic strategies directly targeting fibroblast and immune-modulatory pathways. FAK is a key mediator of extracellular matrix remodeling, CAF activation, and suppression of antitumor immunity and is frequently upregulated in PDAC^[101]^. Preclinical studies indicate that FAK inhibition reduces desmoplastic matrix deposition, enhances CD8+ T-cell infiltration, and sensitizes PDAC tumors to immune checkpoint inhibitors and radiotherapy^[102]^. Consistent with these findings, early clinical data from a neoadjuvant and adjuvant study in high-risk resectable PDAC (NCT03727880) demonstrate that the combination of the FAK inhibitor defactinib with pembrolizumab is associated with reduced fibroblast infiltration and increased signatures of M1 macrophages and CD8+ T-cells compared with pembrolizumab alone^[21]^. However, persistent CXCR4 pathway activation in this setting suggests the emergence of compensatory stromal-mediated resistance, highlighting this compensatory mechanism and providing a rationale for investigating combined FAK and CXCR4 targeting as a strategy for more durable TME reprogramming^[21]^.

Beyond restoring antitumor immunity, FAK inhibition may also overcome stromal-mediated resistance by suppressing CAF-derived paracrine signaling, including FGF1-dependent activation of MYC, further supporting FAK inhibition as a strategy to potentially enhance KRAS inhibitor efficacy^[99]^.

Altogether, evidence derived from PDAC preclinical models and clinical studies indicates that the PDAC stroma functions not only as a physical barrier but also as a dynamic signaling compartment that sustains tumor survival, restricts immune infiltration, maintains downstream MAPK and PI3K signaling through RTK-dependent bypass mechanisms, and promotes adaptive transcriptional programs, including MYC and YAP1, even in the presence of potent KRAS inhibitors. These preclinical and translational findings suggest that strategies aimed at modulating stromal composition, disrupting CAF–tumor paracrine signaling, and relieving immune exclusion may complement KRAS-directed therapies and potentially contribute to more durable pathway suppression and antitumor responses. However, clinical experience with broad stromal-remodeling approaches in PDAC has so far shown limited benefit, underscoring the need to define which stromal, paracrine, and immune components are therapeutically actionable in the setting of KRAS-targeted treatment.

## THERAPEUTIC STRATEGIES TO OVERCOME RESISTANCE TO KRAS INHIBITORS

Collectively, the resistance mechanisms described indicate that durable benefit from KRAS inhibition in PDAC will likely require combination strategies that simultaneously constrain on-target adaptation, prevent network-level bypass signaling, suppress downstream pathway rebound, decrease microenvironment-mediated protection, and deepen and improve the durability of KRAS pathway suppression.

Notably, the clinical success of these approaches will likely depend not only on biological rationale but also on biomarker-guided patient selection, optimized sequencing, and strategies that preserve an acceptable therapeutic index.

Importantly, resistance in PDAC rarely arises from one alteration^[103]^; instead, PDAC tumors tend to evolve into complex, layered adaptive states under therapeutic pressure. As a result, current drug development is increasingly focused on rational combinations designed to reduce pathway plasticity and limit the emergence of drug-tolerant persistent populations, while remaining tolerable in a disease setting where baseline performance status and chemotherapy-related toxicity often constrain treatment intensity.

KRAS-dependent resistance may involve secondary *KRAS* alterations, increased KRAS dosage, or accelerated GDP-GTP cycling^[15,60,81]^. Thus, one therapeutic concept is to reduce the capacity of tumor cells to regenerate active RAS signaling upstream of KRAS. This rationale uses combinations that inhibit KRAS together with regulators of nucleotide exchange and RAS activation, including SOS1 and SHP2. Preclinical data from studies by Fedele *et al*.^[54]^ and Hofmann *et al.*^[53]^ demonstrate that inhibition of SHP2 and SOS1 disrupts KRAS nucleotide cycling and creates a state in which MEK inhibitors become more effective. Several ongoing trials on advanced solid tumors, mainly in NSCLC, are directly using this approach, including the SOS inhibitor BI1701963 combined with trametinib (NCT04111458) and the SOS1 inhibitor MRTX0902 combined with adagrasib (NCT05578092). SHP2 blockade is similarly being tested to suppress adaptive RAS activation in combination with KRAS inhibition, for example, adagrasib with TNO155 in the KRYSTAL-2 trial in solid tumors, including PDAC (NCT04330664) and JDQ443 combined with TNO155 in advanced solid tumors, mainly in lung tumors (NCT04699188).

**Figure 5:**
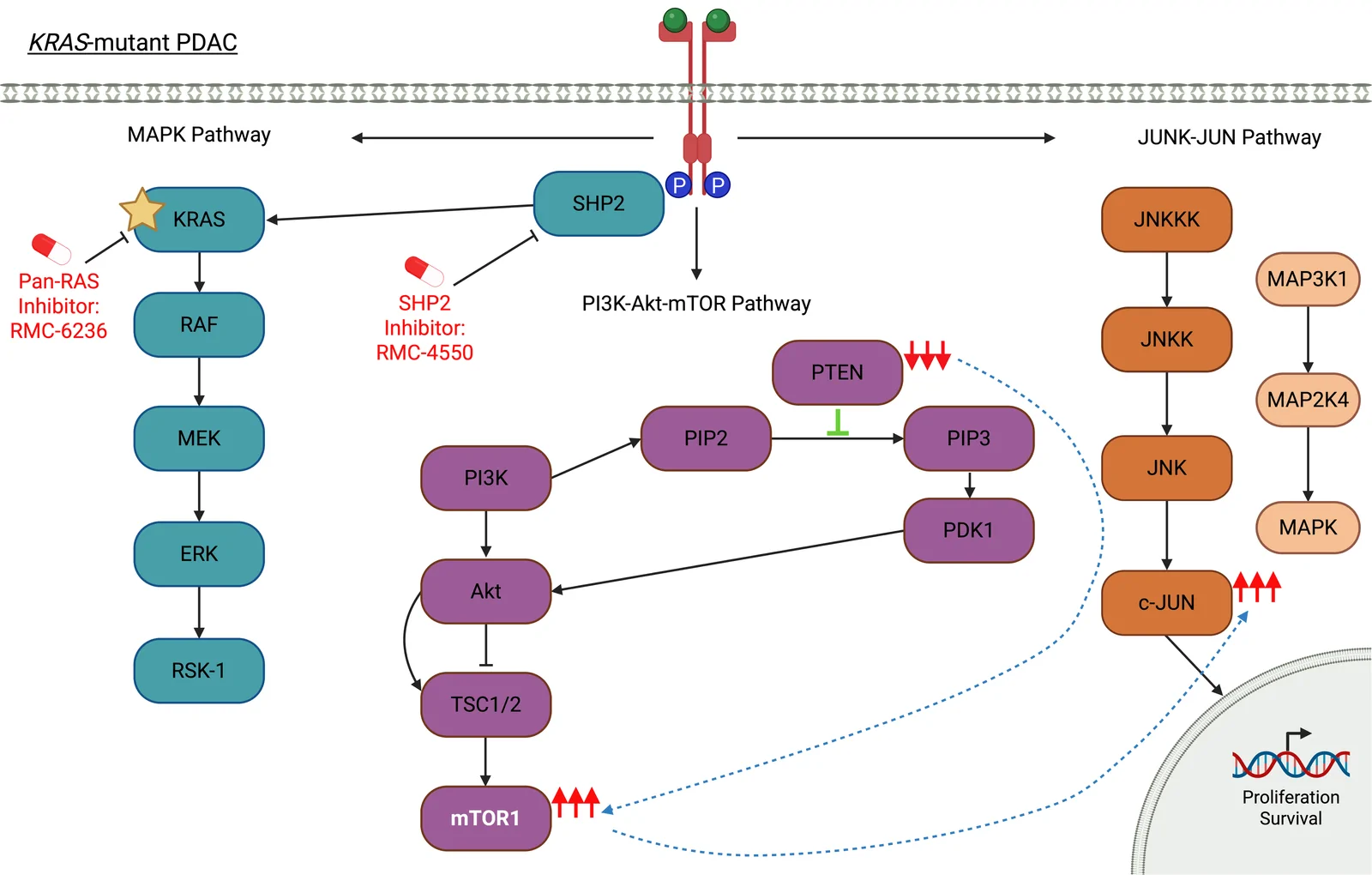
Graphical overview illustrating how KRAS-mutant PDAC cells switch dependency to parallel survival pathways when exposed to combination strategies targeting both KRAS and upstream regulators within the MAPK pathway. For this specific dual-inhibitor combination targeting SHP2 and RAS, resistance emerges through loss of PTEN, a negative regulator of the PI3K–AKT–mTOR axis. This results in overactivation of the PI3K–AKT–mTOR signaling pathway, causing elevated mTOR levels that ultimately drive c-JUN upregulation. Subsequently, overactivation of c-JUN promotes proliferation and survival of resistant cells under sustained inhibition of RAS signaling^[104]^. Created in BioRender. Giovannetti, E. (2026) https://BioRender.com/xn2tx7r. PDAC: Pancreatic ductal adenocarcinoma; MAPK: mitogen-activated protein kinase; SHP2: Src homology 2 domain-containing protein tyrosine phosphatase 2; RAS: RAS family of small GTPases; PTEN: phosphatase and tensin homolog; PI3K: phosphoinositide 3-kinase; AKT: AKT serine/threonine kinase; mTOR: mechanistic target of rapamycin; JUN: Jun proto-oncogene; RAF: rapidly accelerated fibrosarcoma; MEK: mitogen-activated protein kinase kinase; ERK: extracellular signal-regulated kinase; RSK-1: ribosomal protein S6 kinase 1; PIP: phosphatidylinositol phosphate; PDK1: 3-phosphoinositide-dependent protein kinase 1; TSC1/2: tuberous sclerosis complex 1/2; JUNK: Jun N-terminal kinase; JNK: c-Jun N-terminal kinase; JNKK: JNK kinase; JNKKK: JNK kinase kinase; MAP3K1: mitogen-activated protein kinase kinase kinase 1; MAP2K4: mitogen-activated protein kinase kinase 4.

However, more recent data indicate that overexpression of alternative genes within this complex network can sustain tumor cell proliferation even in the presence of combination therapies targeting the same axis, SHP2, and the RAS-MEK-ERK pathway. A recent *in vitro* study provided preliminary evidence that, although the combination of a pan-RAS(ON) inhibitor and a SHP2 inhibitor has synergistic effects, resistant cell populations may emerge and display JUN overexpression [Figure 5]. In particular, resistant cells retain their proliferative capacity even under continuous pharmacological pressure and despite the effective suppression of ERK signaling, suggesting partial escape from KRAS-ERK dependency^[104]^. Although clinical validation is still lacking, these findings support the possibility that dual targeting of KRAS and upstream MAPK regulators may be bypassed through adaptive transcriptional rewiring, potentially involving PI3K–AKT–mTOR signaling and JUN-dependent survival programs.

A translational priority is defining which tumors are primarily limited by RAS cycling *vs.* those dominated by other resistance mechanisms, since the clinical utility of vertical suppression is likely dependent on baseline RTK drive, GEF dependency, and the extent of residual wild-type RAS signaling. These observations further support biomarker-guided selection of combination therapies, allowing treatment to be tailored according to the predominant resistance mechanism while minimizing unnecessary toxicity associated with empiric multi-agent approaches.

From a therapeutic perspective, KRAS-independent bypass signaling provides a rationale for co-targeting compensatory receptor inputs and parallel growth signals: even when KRAS inhibition is pharmacologically “on target”, tumor cells can route around this inhibition by upregulating compensatory RTK signaling and relying more heavily on wild-type RAS isoforms, which may be sufficient to rebuild MAPK and PI3K pathway output^[64]^. That logic naturally points toward combination regimens that shut down the most relevant receptor inputs and parallel growth signals, rather than assuming that KRAS monotherapy will provide sustained disease control. This rationale has motivated the clinical investigation of biomarker-informed co-targeting therapies, most commonly pairing KRAS inhibitors with receptor- or ligand-directed approaches.

Consistent with this network-level model of resistance, several KRAS G12C trial programs already include EGFR-directed components, such as adagrasib combined with cetuximab or afatinib (NCT03785249), divarasib combinations that include cetuximab or erlotinib (NCT04449874), and sotorasib in combination with panitumumab (NCT05198934). A major challenge, therefore, is not the lack of rational combination strategies, but rather the limited ability to predict, at the individual patient level, which compensatory signaling nodes will predominate and how these dependencies dynamically evolve under therapeutic selection pressure. This limitation underscores the importance of longitudinal molecular profiling^[18,81]^. Because serial tumor sampling is often not feasible, plasma-based monitoring (e.g., circulating tumor DNA or extracellular vesicle analyses) might offer a practical approach to detect emergent RTK amplifications or broader signaling reprogramming at an early stage, thereby enabling adaptive therapeutic strategies rather than interventions initiated only after overt clinical progression^[105]^.

Downstream pathway reactivation is another key obstacle; signaling output can recover even when KRAS and its proximal activators are effectively inhibited. This keeps the RAF–MEK–ERK and PI3K–AKT–mTOR cascades central to resistance-oriented therapeutic strategies. Current therapeutic development is therefore pursuing complementary strategies aimed at limiting compensatory survival mechanisms and preventing downstream pathway reactivation, with approaches progressing to different stages of clinical and preclinical evaluation.

First, downstream inhibitors are combined with agents that suppress compensatory survival mechanisms such as autophagy, which is induced by MAPK inhibition in PDAC^[80,81]^. This is being tested clinically with binimetinib plus hydroxychloroquine (NCT04132505), trametinib plus hydroxychloroquine in the THREAD study (NCT03825289), and other hydroxychloroquine-based combinations with MEK or ERK inhibitors (NCT06229340) [Table 4]. These approaches are intended to limit adaptive survival mechanisms rather than intensify MAPK blockade.

**Table 4:** Clinical trials of agents targeting upstream regulators, downstream effectors, and related components of the KRAS signaling pathway

**ClinicalTrials.gov ID**	**Phase**	**Status**	**Enrollment**	**Treatment arm(s)**	**Mechanism of action**	**Combination regimen**	**RAS mutation status**	**Cohort**	**Primary outcome/end point**
Upstream inhibitors									
NCT04111458	I	Active, not recruiting	71 - Actual	BI1701963	SOS1 inhibitor	Trametinib	KRAS, any variant	Advanced solid tumors including PDAC	DLTs, ORR
NCT05578092	I/II	Terminated	64 - Actual	MRTX0902	SOS1 inhibitor	Adagrasib (MRTX849)	KRAS, any variant (Monotherapy) - KRAS G12C (Combination)	Advanced solid tumors including PDAC	DLTs, AEs, ORR, PFS, OS
NCT04330664 (KRYSTAL-2)	I	Completed	86 - Actual	Batoprotafib (TNO155)	SHP2 inhibitor	Adagrasib (MRTX849)	KRAS G12C	Advanced solid tumors including PDAC	AEs
NCT03634982	I	Unknown status	133 - Actual	Vociprotafib (RMC-463)	SHP2 inhibitor	N/A	KRAS amplifications, KRAS G12C (Subprotocol 2)	Relapsed/refractory solid tumors including PDAC	AEs, DLTs
NCT06026410	I	Recruiting	300 - Estimated	Darlifarnib (KO-2806)	Farnesyltransferase inhibitor	Cabozantinib, Adagrasib (MRTX849)	HRAS/KRAS-mutant and/or amplified	Advanced solid tumors including PDAC	DLTs, AEs, ORR
Downstream inhibitors									
NCT04132505	I	Completed	34 - Actual	Binimetinib	MEK inhibitor	Hydroxychloroquine	KRAS, any variant	Metastatic PDAC	MTD
NCT05554367 (Combo-MATCH)	II	Active, not recruiting	199 - Estimated	Binimetinib	MEK inhibitor	Palbociclib	KRAS, HRAS, NRAS mutations and/or RAF alterations	Advanced solid tumors including PDAC	ORR
NCT03825289 (THREAD)	I	Terminated	25 - Actual	Trametinib	MEK inhibitor	Hydroxychloroquine	N/A	Stage II to IV PDAC	DLTs
NCT06229340 (NTO-RAS)	II	Recruiting	20 - Estimated	Trametinib, Cobimetinib, or Binimetinib	MEK inhibitors	Hydroxychloroquine, bevacizumab	KRAS, HRAS, NRAS mutations	Refractory malignancies including PDAC	ORR
NCT05585320	I/II	Active, not recruiting	209 - Actual	Atebimetinib (IMM-1-104)	MEK inhibitor	Gemcitabine/nab-paclitaxel, mFOLFIRINOX	KRAS, NRAS, or HRAS activating mutations	Advanced solid tumors including PDAC	AEs, DLTs, ORR
NCT05669482	I/II	Active, not recruiting	40 - Estimated	Avutometinib (VS-6766)	RAF/MEK clamp	Defactinib, gemcitabine and nab-paclitaxel	KRAS activating mutation	Metastatic PDAC	DLTs, ORR

RAS: RAS family of small GTPases; SOS1: son of sevenless 1; PDAC: pancreatic ductal adenocarcinoma; DLTs: dose-limiting toxicities; ORR: objective response rate; AEs: adverse events; PFS: progression-free survival; OS: overall survival; SHP2: Src homology 2 domain-containing protein tyrosine phosphatase 2; N/A: not applicable; MEK: mitogen-activated protein kinase kinase; MTD: maximum tolerated dose; RAF: rapidly accelerated fibrosarcoma.

Nonetheless, in the case of binimetinib plus hydroxychloroquine, a challenging toxicity profile and limited clinical activity were observed in patients with chemorefractory metastatic PDAC, further highlighting that promising strategies may prove infeasible in PDAC patients, where poor performance status can impair drug tolerance^[106]^.

A complementary strategy is to target metabolic adaptations that sustain tumor fitness despite pathway inhibition. Glutamine metabolism represents a particularly relevant vulnerability, as KRAS-driven glutamine utilization supports anabolic and redox homeostasis in PDAC. Although GLS inhibition alone has shown limited efficacy, broader disruption of glutamine metabolism can enhance therapeutic activity when combined with inhibition of compensatory pathways. For example, glutamine antagonism with DON can be potentiated by L-asparaginase, while depletion of asparagine has also been shown to enhance the effects of MEK inhibition in preclinical PDAC models^[83,84]^. These findings suggest that metabolic dependencies can be therapeutically exploited to prevent compensatory signaling and survival following MAPK pathway inhibition. Similarly, KRAS-driven alterations in lipid storage and utilization, including suppression of HSL and increased lipid-droplet accumulation, create additional metabolic vulnerabilities that contribute to invasive and metastatic behavior^[83]^. Although these lipid metabolic dependencies have not yet been directly established as mechanisms of resistance to KRAS inhibitors, their disruption represents a potential complementary approach for limiting tumor adaptation and progression.

Second, recognition of feedback MAPK reactivation has driven multi-node inhibition strategies^[79]^. RAF–MEK clamp approaches are designed to prevent pathway rebound by targeting both signaling and feedback loops. In PDAC, this is exemplified by avutometinib (VS-6766) combined with defactinib and chemotherapy in metastatic disease (NCT05669482).

In pancreatic cancer, preliminary preclinical evidence supports dual inhibition of KRAS and PI3K. In orthotopic models, the combination of a KRAS G12D inhibitor, MRTX1133, and a dual BRD4/PI3K inhibitor reduced tumor growth^[107]^. In addition, *in vitro*, the use of the PI3K inhibitor re-sensitized cell lines resistant to MRTX1133^[107]^. In keeping with these preclinical findings, PI3K pathway co-targeting is now being explored clinically by the evaluation of divarasib in combination with the PI3K inhibitor inavolisib (NCT04449874).

YAP/TAZ signaling has also been implicated as a key driver of resistance to targeted therapies^[44,45]^. Accordingly, emerging preclinical data suggest that epigenetic therapies may provide a potential strategy to overcome resistance in KRAS-mutant cancer, including PDAC, by indirectly targeting YAP/TAZ signaling. In this context, combining a class I histone deacetylase (HDAC) inhibitor with KRAS/MAPK inhibitors can enhance antitumor effects *in vitro* and *in vivo*, highlighting a tight functional interplay between HDAC1, HDAC2, HDAC3, and RAS/MAPK signaling^[108]^. Mechanistic analyses have revealed that class I HDAC inhibitors exert profound effects on the nonhistone proteins p53 and c-Myc. In turn, p53 modulation indirectly affects the transcriptional coactivators YAP and TAZ within the Hippo pathway, ultimately increasing tumor vulnerability in a context-dependent manner^[108]^.

A key limitation across these downstream strategies is the therapeutic index. Although deeper suppression of oncogenic signaling may improve tumor control, simultaneous inhibition of multiple signaling nodes also increases the risk of cumulative systemic toxicity, potentially limiting dose intensity and treatment duration. Consequently, defining pharmacodynamic thresholds that achieve sufficient tumor pathway inhibition while preserving tolerability will be essential for successful clinical translation.

Addressing this gap will be critical for translating downstream pathway combinations into durable clinical benefits in KRAS-driven PDAC.

Of note, enhancing the efficacy and durability of KRAS inhibitors may not rely only on reinforcing the blockade of oncogenic signaling. Rather than focusing exclusively on inhibiting pathway reactivation or KRAS-dependent escape mechanisms, other approaches aim to amplify the impact of KRAS inhibition by exploiting collateral cellular states induced by oncogene suppression. In PDAC, this requires targeting key processes regulated by KRAS, including cell cycle control, DNA damage repair capacity, apoptosis induction, and metabolic homeostasis^[80,81,107,109-111]^.

Based on previous evidence that the combined inhibition of the MEK-ERK and CDK4 pathways produces synergistic antitumor effects, a recent study has shown that the same synergy can be achieved in PDAC by combining direct RAS inhibitors with the CDK4 inhibitor palbociclib^[109]^. *In vitro*, this combination significantly increased susceptibility to prolonged cell-cycle arrest, thereby enhancing the antiproliferative effects of RAS inhibition. However, *in vivo*, this synergy was not fully replicated, likely due to adaptive changes in the TME, including increased vascularization. Nevertheless, KRAS–CDK4 co-inhibition represents a potential therapeutic strategy that warrants further investigation as a means of overcoming or delaying resistance to RAS-targeted therapies^[109]^.

In addition to combined strategies based on direct cell-cycle inhibition, various approaches exploit DNA repair defects arising from oncogene-induced alterations in cell-cycle control. In PDAC, inhibition of KRAS or downstream MAPK signaling can impair homologous recombination, thereby sensitizing cells to poly ADP-ribose polymerase (PARP) inhibitors^[110]^. In particular, a recent study demonstrated that direct inhibition of KRAS G12D induces an even more profound suppression of homologous recombination through the transcriptional downregulation of key DNA repair genes, partly independent of cell cycle arrest and inhibition of the MAPK pathway^[110]^. This allele-specific, cell-cycle-independent branch of homologous recombination regulation persists even with adaptive reactivation of downstream signaling, supporting the investigation of KRAS G12D–PARP co-targeting as a potential strategy to overcome or delay resistance^[110]^.

Another study demonstrated that, in PDAC, the activity of single-agent KRAS inhibitors can be enhanced by exploiting vulnerabilities linked to pro-death signaling and survival mechanisms, rather than by targeting the reactivation of oncogenic signaling pathways^[111]^. In this work, the KRAS G12D inhibitor MRTX1133 was combined with the BCL-xL degrader DT2216 and the mTOR inhibitor everolimus. The triple combination targeted key anti-apoptotic proteins identified as major determinants of the limited efficacy of MRTX1133, a compound characterized by predominantly cytostatic activity. This approach not only increased apoptosis induction but also showed antitumor activity in preclinical models treated with MRTX1133, highlighting how the elimination of adaptive survival programs may represent a rational approach to overcoming resistance to KRAS inhibitors^[111]^.

TME-mediated resistance mechanisms support the idea that the PDAC stroma functions not only as a physical impediment to drug delivery, but also as an active signaling compartment that preserves tumor cell survival and constrains immune-mediated tumor control during KRAS inhibition^[95]^. Consequently, contemporary resistance-prevention strategies increasingly extend beyond tumor-intrinsic targeting to incorporate stromal and immune-modulatory interventions. Among currently investigated stromal-targeting strategies, FAK inhibition has progressed into clinical evaluation. The incorporation of defactinib into combination regimens, including its use with the RAF-MEK clamp avutometinib in PDAC (NCT05669482), is based on the rationale that FAK inhibition may attenuate adhesion-dependent survival signaling, alleviate immune exclusion, and potentially potentiate suppression of tumor-intrinsic oncogenic pathways. These approaches aim to reprogram, rather than ablate, key stromal signaling circuits that contribute to therapeutic resistance. Another major unresolved challenge in this domain is the substantial heterogeneity of CAF subtypes within PDAC^[96]^. Non-selective modulation of the stroma risks inducing compensatory programs or promoting alternative tumor-supportive states, thereby limiting durable therapeutic benefit. Future progress will require the development of robust biomarkers that define stromal states, improved spatial sampling and analysis strategies, and mechanistic clinical trials that integrate stromal and immune parameters as primary endpoints instead of supplementary correlations of response.

Lastly, overcoming resistance to KRAS inhibitors also requires accurate evaluation and optimization of pharmacokinetic and pharmacodynamic profiles, with the aim of achieving a deep and sustained inhibition of the target at the tumor level. For example, these aspects were investigated in the phase I study of setidegrasib (ASP3082), a selective degrader of KRAS G12D, in patients with NSCLC and PDAC^[112]^. From a clinical perspective, preliminary signs of antitumor activity were observed, with dose-dependent degradation of the KRAS protein and inhibition of downstream signaling pathways. The recommended phase II dose was identified as 600 mg administered intravenously once weekly, based on the balance between pharmacokinetic exposure, pharmacodynamic activity, and tolerability. Despite responses in a subset of patients, the duration of observed benefit was limited in the available early clinical data. These findings suggest that, despite effective on-target modulation, resistance mechanisms may still emerge, potentially driven by activation of bypass signaling pathways rather than insufficient pharmacodynamic inhibition of the target^[112]^.

Across all combination strategies, three gaps limit the translation into the clinic. First, resistance mechanisms are likely to differ by KRAS allele, baseline transcriptomic subtype, and microenvironmental context, yet patient selection in many early-phase trials remains broad. Second, the field still lacks consensus on how to sequence combinations: whether to start with multi-agent regimens to prevent clonal escape, or to reserve combination therapy for molecularly defined progression states detected by longitudinal monitoring^[18,113]^. Third, the optimal dosing strategy remains undefined. Sustained target suppression may maximize pathway inhibition but may also narrow the therapeutic index by increasing cumulative toxicity and selective pressure for adaptive resistance. Rational intermittent dosing schedules that maintain sufficient pharmacodynamic inhibition while allowing recovery of normal tissues deserve prospective evaluation^[47,113]^.

These limitations point to the need for adaptive trial designs that integrate serial ctDNA profiling, treatment biopsies when feasible, and robust pharmacodynamic markers of downstream pathway suppression.

The success of such adaptive strategies will largely depend on identifying and validating robust predictive and pharmacodynamic biomarkers. Beyond the presence of a KRAS mutation, the specific KRAS allele (e.g., G12C, G12D, G12V, G12R) may influence therapeutic response and determine distinct evolutionary trajectories of acquired resistance, supporting allele-specific patient stratification^[15,49,50]^. A notable example is KRAS G12R, which exhibits distinct effector engagement and differential dependencies on intracellular signaling networks compared with other common KRAS alleles. KRAS G12R is unable to interact with the PI3Kα (p110α) effector, resulting in reduced activation of the canonical PI3K-AKT pathway. In PDAC, this defect appears to be partially compensated by PI3Kγ (p110γ) signaling, leading to altered regulation of macropinocytosis and distinct signaling and autophagy dependencies. These biological differences may contribute to unique therapeutic vulnerabilities and potentially distinct resistance paths compared with other KRAS-mutant subtypes, supporting the development of allele-specific therapeutic strategies^[28]^.

Longitudinal assessment of ctDNA, including KRAS-mutant allele fraction and the emergence of KRAS copy-number gain or secondary resistance alterations, could enable early detection of molecular progression and guide timely therapeutic adaptation^[15,18,49-52,60]^. In parallel, pharmacodynamic biomarkers such as suppression of ERK phosphorylation (pERK) may provide direct evidence of effective MAPK pathway inhibition, whereas activation of RTK signaling may indicate pathway reactivation despite continued treatment^[49,60-62]^.

Furthermore, transcriptomic signatures associated with EMT or YAP/TAZ activation, together with stromal characteristics of the TME, may identify adaptive cell states linked to drug tolerance and help prioritize rational combination therapies^[42-46,95]^. Integrating these complementary biomarkers into prospective clinical trials may improve patient selection, enable real-time monitoring of treatment response, and facilitate biomarker-guided therapeutic interventions throughout disease evolution.

**Figure 6:**
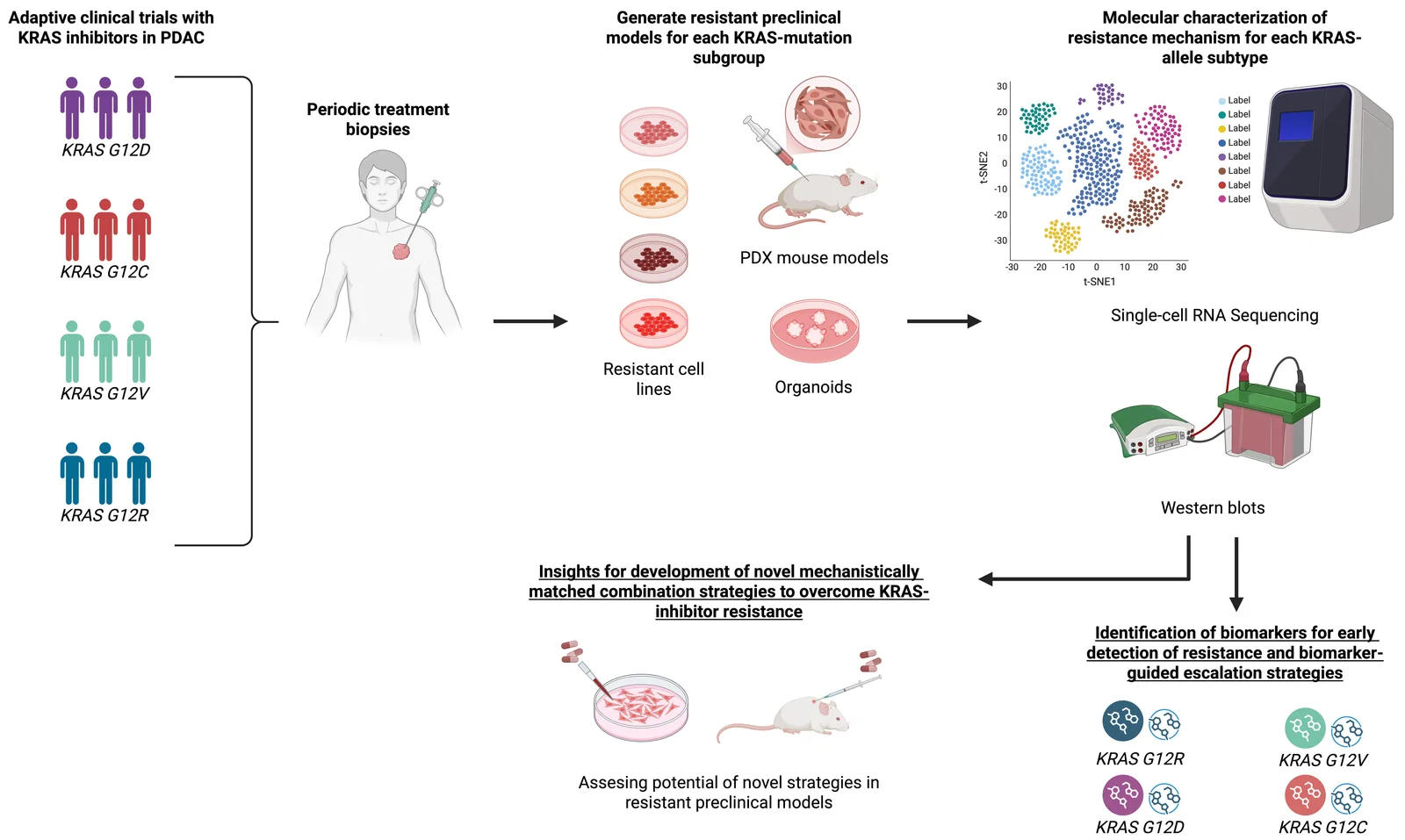
Schematic overview of a workflow that can be applied to establish PDAC-specific models, which are essential for extensive molecular characterization of resistance mechanisms to KRAS inhibitors. Adaptive clinical trials with longitudinal monitoring allow frequent treatment biopsies at several stages of disease progression. These biopsies can be used to develop a variety of resistant preclinical models. Subsequently, scRNA sequencing and western blots can be applied to uncover key drivers of resistance to KRAS inhibition therapies. This approach could ultimately provide crucial insights into vulnerabilities for developing novel therapeutic strategies. Moreover, this workflow could unveil biomarkers for resistance monitoring and biomarker-guided escalation strategies when an escape state is detected. Created in BioRender. Giovannetti, E. (2026) https://BioRender.com/m0pgua5. PDAC: Pancreatic ductal adenocarcinoma; scRNA: single-cell RNA; PDX: patient-derived xenograft; t-SNE: t-distributed stochastic neighbor embedding.

Such adaptive trials with longitudinal monitoring could also help establish PDAC-specific preclinical models that represent each KRAS mutation type. This would be useful for extensive characterization of molecular mechanisms of resistance to KRAS inhibitors and identifying their associated biomarkers [Figure 6]. Specifically, this KRAS allele-specific subgrouping approach could identify predictive biomarkers for early detection of resistance development in patients. This will enable biomarker-guided selection of the most suitable combination therapy for each stage of resistance evolution during clinical trials and adapting the therapeutic approach when escape states are detected. Moreover, PDAC-specific preclinical models can be used to uncover key drivers of resistance to a variety of KRAS inhibiting strategies, which might provide insight into vulnerabilities that could be exploited in novel personalized treatments tailored to KRAS allele-specific resistance mechanisms [Figure 6].

Ultimately, overcoming resistance to KRAS inhibition in PDAC will require mechanistically matched combinations, earlier detection of escape states, and biomarker-guided escalation strategies that balance pathway depth with tolerability.

## CONCLUSION AND FUTURE PERSPECTIVES

Direct inhibition of KRAS represents a rapidly expanding field of research, as KRAS functions as a driver oncogene in several malignancies, including PDAC. Although initially considered “undruggable”, multiple drugs capable of binding and targeting specific KRAS mutations have been developed in recent years, with applications across different KRAS-driven tumors^[9,10]^. The first inhibitors targeting the G12C mutation were sotorasib and adagrasib. More recently, novel compounds have emerged that can target other KRAS mutations, including G12D (e.g., MRTX1133) or multiple mutations simultaneously, such as the pan-KRAS(OFF) inhibitor BI-2865 and the pan-RAS(ON) inhibitor RMC-6236. Although these agents are not yet approved for the treatment of PDAC, they represent promising therapeutic options currently under clinical evaluation^[27,31,114-116]^.

Overall, the evidence discussed in this review indicates that direct inhibition of KRAS in PDAC represents a fundamental therapeutic advance, but in most cases does not translate into durable disease control when used as monotherapy. PDAC emerges as a malignancy characterized by a multilayered architecture of resistance, in which tumor-intrinsic adaptations, redundant signaling networks, and the TME can limit both the intensity and the duration of response^[103]^. However, resistance mechanisms to (K)RAS inhibitors in PDAC have not been systematically evaluated or integrated, and this topic remains far less explored than in other solid tumors.

**Figure 7:**
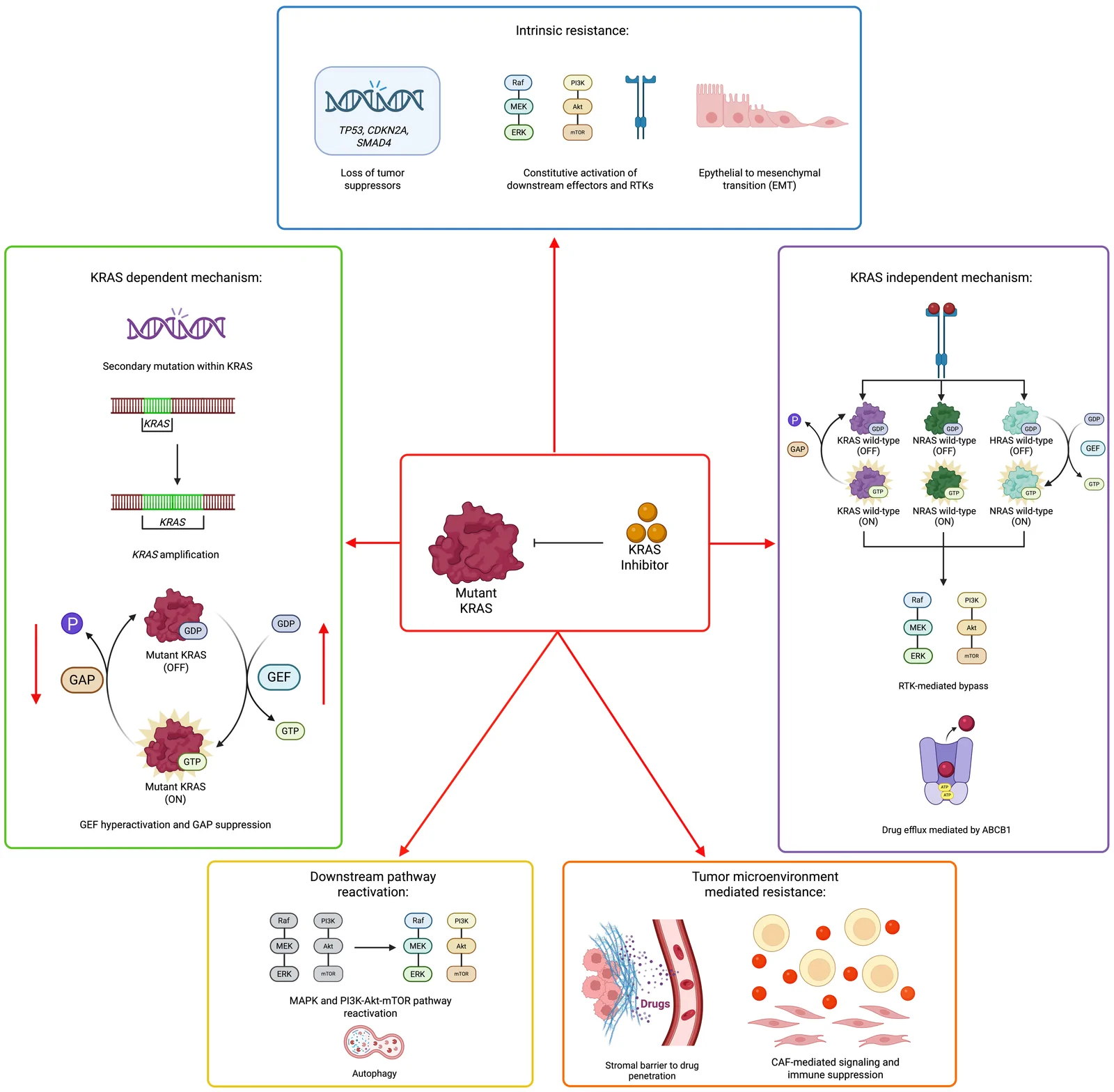
Schematic overview of the mechanisms of resistance to KRAS inhibitors in PDAC. Resistance mechanisms are categorized as intrinsic or acquired and include KRAS-dependent alterations (e.g., secondary mutations or amplification of KRAS), KRAS-independent signaling bypass through RTK activation or amplification (e.g., EGFR, MET), reactivation of downstream pathways (including MAPK and PI3K signaling), and TME-mediated mechanisms such as stromal remodeling and immune modulation. These processes collectively sustain tumor cell survival and proliferation despite pharmacological inhibition of KRAS. Created in BioRender. Giovannetti, E. (2026) https://BioRender.com/c2u0n36. PDAC: Pancreatic ductal adenocarcinoma; RTK: receptor tyrosine kinase; EGFR: epidermal growth factor receptor; MET: mesenchymal-epithelial transition factor; MAPK: mitogen-activated protein kinase; PI3K: phosphoinositide 3-kinase; TME: tumor microenvironment; MEK: mitogen-activated protein kinase kinase; ERK: extracellular signal-regulated kinase; Akt: AKT serine/threonine kinase; mTOR: mechanistic target of rapamycin; GAP: GTPase-activating protein; GDP: guanosine diphosphate; GTP: guanosine triphosphate; GEF: guanine nucleotide exchange factor; ABCB1: ATP-binding cassette subfamily B member 1; CAF: cancer-associated fibroblast.

One key point emerging from both preclinical and clinical data is that resistance in PDAC rarely converges on a single leading mechanism. Multiple resistance mechanisms have been documented [Figure 7], including KRAS-dependent mechanisms (secondary mutations or gene amplification), KRAS-independent bypass pathways driven by RTKs, reactivation of the MAPK and PI3K–AKT–mTOR pathways, active remodeling of the TME, and metabolic reprogramming^[39,60,74,85]^. This stratified resistance landscape suggests that, in advanced PDAC, KRAS functions as a central node within a highly plastic network capable of rapidly redistributing oncogenic signaling under pharmacological pressure.

Within this context, transcriptional plasticity emerges as a critical determinant of the limited response to KRAS inhibitors. Activation of YAP/TAZ-dependent programs associated with EMT, stem-like properties, and reduced dependency on MAPK signaling contributes to both primary and acquired resistance in PDAC^[43,44,46]^. Recent studies further indicate that YAP1 reactivation is not confined to tumor cells but is accompanied by immunosuppressive remodeling of the TME during prolonged treatment with RAS inhibitors, suggesting functional cooperation between tumor-intrinsic adaptation and the TME^[95]^. These initial studies describe a phenomenon that has, until now, been observed only in a fragmented and partial manner. Results from clinical trials involving large patient cohorts, together with systematic analyses of multiple preclinical studies, are required to construct a more comprehensive and representative framework of resistance to RAS inhibitors in PDAC.

Future perspectives must therefore be based on a rational integration of therapeutic strategies, rather than on intensifying single signaling axes. The most promising combinations are those designed to simultaneously target distinct yet interconnected nodes of the resistance network, such as KRAS and regulators of GTP/GDP cycling (SHP2, GEF and GAP proteins)^[53,54]^, KRAS and major RTK-driven bypass pathways^[60]^ or KRAS and adaptive survival programs such as autophagy and apoptosis. However, available data indicate that even well-rationalized vertical combinations can be bypassed through the selection of alternative transcriptional programs, as demonstrated by the emergence of JUN-dependent states in resistant models^[104]^.

The TME actively determines resistance, so combination strategies must extend beyond the tumor cell. Observations that KRAS inhibition can initially convert the TME into a more immunostimulatory state, followed by a subsequent shift toward immunosuppression, suggest the existence of a temporal therapeutic window for integration with immunotherapy or stromal-targeting approaches^[95,100]^. However, clinical exploitation of this window requires a more refined understanding of this phenomenon.

In conclusion, as KRAS inhibitor monotherapy becomes more widely implemented in PDAC, tumors rapidly reprogram their signaling networks and remodel the TME in response to therapeutic pressure. This adaptive plasticity represents a major barrier to sustained therapeutic efficacy. Overcoming such resilience will require the development of rational, mechanism-based combination strategies aimed at converting inhibition of KRAS from a transient response into durable disease control, ultimately improving clinical outcomes in PDAC and shaping the future of therapy for one of the most refractory malignancies in oncology.

## DECLARATIONS

### AI and AI-assisted tools statement

During the preparation of this manuscript, the AI tools, GPT and Copilot (based on version GPT-5.5 released 2025-08-07 and subsequent versions) was used solely for language editing. The tool did not influence the study design, data collection, analysis, interpretation, or the scientific content of the work. All authors take full responsibility for the accuracy, integrity, and final content of the manuscript.

### Copyright

© The Author(s) 2026.
